# Redox regulation of EGFR steers migration of hypoxic mammary cells towards oxygen

**DOI:** 10.1038/s41467-018-06988-3

**Published:** 2018-10-31

**Authors:** Mathieu Deygas, Rudy Gadet, Germain Gillet, Ruth Rimokh, Philippe Gonzalo, Ivan Mikaelian

**Affiliations:** 1 Université de Lyon, Université Claude Bernard Lyon 1, INSERM 1052, CNRS 5286, Centre Léon Bérard, Centre de recherche en cancérologie de Lyon, 69373 Lyon, France; 2 Hospices civils de Lyon, Laboratoire d’anatomie et cytologie pathologiques, Centre Hospitalier Lyon Sud, Chemin du Grand Revoyet, 69495 Pierre Benite, France; 3 Laboratoire de Biochimie, Faculté de médecine de Saint-Etienne, CHU de Saint-Etienne, 42000 Saint-Etienne, France

## Abstract

Aerotaxis or chemotaxis to oxygen was described in bacteria 130 years ago. In eukaryotes, the main adaptation to hypoxia currently described relies on HIF transcription factors. To investigate whether aerotaxis is conserved in higher eukaryotes, an approach based on the self-generation of hypoxia after cell confinement was developed. We show that epithelial cells from various tissues migrate with an extreme directionality towards oxygen to escape hypoxia, independently of the HIF pathway. We provide evidence that, concomitant to the oxygen gradient, a gradient of reactive oxygen species (ROS) develops under confinement and that antioxidants dampen aerotaxis. Finally, we establish that in mammary cells, EGF receptor, the activity of which is potentiated by ROS and inhibited by hypoxia, represents the molecular target that guides hypoxic cells to oxygen. Our results reveals that aerotaxis is a property of higher eukaryotic cells and proceeds from the conversion of oxygen into ROS.

## Abstract (web summary)

Aerotaxis, chemotaxis towards oxygen, occurs in bacteria and likely in cancer cells. Here the authors find that confined cells from different tissues escape hypoxia by aerotaxis, a process independent of mitochondria and the HIF pathway, and dependent on EGF receptor interpretation of a ROS gradient in mammary cells.

## Introduction

During the course of evolution, oxygen has become essential for most eukaryotic life. As the last acceptor of the mitochondrial electron transport chain, sufficient oxygen availability is required to regenerate ATP. Aerobic organisms mainly rely on mitochondrial respiration for this process. However, excessive oxygen can also fuel the production of potentially deleterious reactive oxygen species. In this regard, migration to an optimal oxygen concentration can be considered as an adaptive mechanism. This process has been demonstrated over a century ago in bacteria and called aerotaxis^1,2^. Recently, the choanoflagellate *Salpingoeca rosetta*, the closest unicellular relative of animals was also shown to display aerotaxis^3^. Most eukaryotic cells contain an adaptive pathway to sense and adapt to low oxygen concentrations, the hypoxia-inducible factor (HIF) pathway. The core component of the HIF system (PHD/HIF/VHL) is present in the simplest known animal, the placozoan *Trichoplax adhaerens*, first appearing in the Precambrian or 550 million years ago^4^. HIF proteins belong to a family of transcription factors that regulate over 200 genes required to counteract hypoxia^5^. The main mechanism supporting the rapid accumulation of HIF in hypoxia, and its degradation in normoxia, depends on oxygen concentration itself, which is sensed by key enzymes, the PHDs. Indeed, in normoxia, PHDs hydroxylate HIFs on key proline residues using oxygen as a substrate. This leads to the creation of a molecular recognition tag for the ubiquitin ligase VHL that ultimately targets these factors for proteasomal degradation^6^.

In multicellular organisms, oxygen levels can fluctuate between 1 and 10% depending on the tissues, owing to the levels of oxygen transport and metabolic consumption. Interestingly, some developmental processes occur in the vicinity of hypoxic regions, suggesting that oxygen is a developmental morphogen^7,8^. One such process is mammalian placentation that connects the rapidly growing but hypoxic foetus to the maternal uterine circulatory system, ensuring nutrient and oxygen supply. This process relies on local hypoxia that was shown to dictate the ability of cytotrophoblast cells to proliferate or to invade the maternal endometrium^9^. The role of hypoxic gradients is also relevant in pathological contexts such as cancer metastasis^10,11^. Within tumours, severe hypoxia resulting from uncontrolled cell proliferation activates the HIF pathway. In turn, HIF accumulation results in neovascularisation through VEGF induction but also triggers the epithelial to mesenchymal transition (EMT), providing cancer cells with migratory and invasive properties, ultimately leading to metastasis^12^. Although recent reports have suggested that cancer cell migration to the neighbouring blood vessels can be oriented by oxygen gradients, the mechanisms and pathways involved in this process remain understudied^13–15^.

Hence, directed migration of cells within hypoxic gradients is of tremendous interest in a number of biological or pathological processes. To address this issue, we designed a new experimental system in which cell migration within self-generated oxygen gradients can be observed, measured and characterised in real-time. Here, we show that hypoxic epithelial cells migrate with a remarkable directionality to regions with higher oxygen concentrations. We demonstrate that aerotactic migration is independent of the HIF/PHD pathway but requires ROS production, which itself requires oxygen as a substrate. Finally, we reveal that aerotaxis proceeds from the redox regulation of EGFR (epithelial growth factor receptor) activity by ROS for mammary epithelial cells.

## Results

### Chemotaxis of epithelial cells towards oxygen

**Fig. 1:**
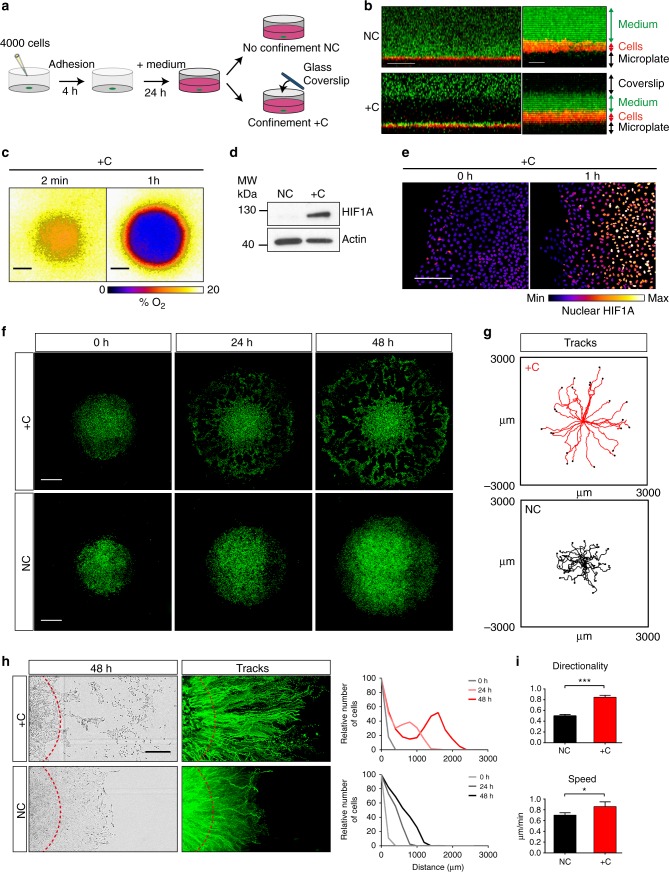
Epithelial cells exposed to deep hypoxia undertake directional migration towards oxygen. **a** Schematic diagram of the cell confinement assay. **b** *Z*-axis confocal analysis of LifeAct-mCherry-expressing MCF10A cells under confinement (+C) or not (NC), showing a layer of ∼7–10 µm of culture medium supplemented with Oregon Green (green) which separates the cells (red) from the coverslip (black). Right panels are magnified regions from left panels. Scale bars, 100 µm (left), 10 µm (right). **c** Detection of hypoxia under confinement with the Visisens camera demonstrating the formation of a steep oxygen gradient at the edge of the cell cluster. Oxygen level is shown in pseudocolors (also see Supplementary Movie 1). **d** Immunoblot showing HIF1A stabilisation after confinement (24 h). Molecular weights (Mw) are indicated in kDa. **e** Hypoxia gradient at the edge of the cluster revealed by HIF1A stabilisation as soon as 1 h after confinement. HIF1A levels are represented in pseudocolors. Scale bar, 200 µm. **f** Representative sequential images of H2B-GFP-expressing MCF10A cells confined (+C) or not confined (NC) showing outwards migration of the cells from the edge of the cluster. Scale bars, 1 mm. **g** Individual tracking of cells (*n* = 24) at the margin of the cell cluster for 48 h, from experiments depicted in (**f**). Tracking is representative of more than three different experiments (also see Supplementary Movies 2–3). **h** Directed migration and redistribution of H2B-GFP-expressing MCF10A cells visualised after 48 h of confinement or unconfined. Left panels: bright field images at 48 h with a red dotted-line indicating the front of the cell cluster at 0 h. Middle panels: tracks of H2B-GFP expressing MCF10A confined or unconfined for 48 h. Right panels: relative distribution of MCF10A cells at the edge of the cluster at 0, 24 and 48 h. Scale bar, 500 µm. **i** Mean values of directionality and speed calculated from (**g**) (mean ± SD; *n* = 3 independent experiments). See Methods for further information about the calculation of motility parameters. ***P < 0.001, *P < 0.05 by two-tailed Student’s *t*-test. NC not confined, +C confined

To investigate whether hypoxic eukaryotic cells could sense oxygen gradients and move directionally to regions of higher oxygen concentration, we developed an assay in which MCF10A immortalized mammary epithelial cells were confined to a small volume of medium, thus generating hypoxic conditions and associated oxygen gradients through mitochondrial respiration (Fig. 1a, see Methods for details). Confocal microscopy confirmed that confined MCF10A cells were not compressed by the coverslip but separated from it by a layer of medium of ∼7–10 µm (Fig. 1b). As expected, cell respiration under confinement rapidly generated deep hypoxia with a steep oxygen gradient at the periphery of the cell cluster, which was visualized by dynamic measurements of oxygen levels with the Visisens detector unit (Fig. 1c, Supplementary Movie 1) and by a marked and rapid accumulation of the HIF1A protein (Fig. 1d–e and supplementary Fig. 1a, b). HIF1A target genes were also upregulated by confinement (Supplementary Fig. 1c). In addition, and as expected for cells exposed to hypoxia, confined cells expressed the typical markers of EMT (Supplementary Fig. 1d). Strikingly, and in contrast with what was observed in unconfined conditions, cells at the border of the cluster started to detach and migrate outwards so as to escape regions of profound hypoxia, as early as 4 h after confinement (Fig. 1f, Supplementary Movies 2–3). This remarkable directionality became obvious by tracking cells located at the margin of the cluster (Fig. 1g) and by detecting cell redistribution at 24 h and 48 h (Fig. 1h). Tracking analysis demonstrated that confined cells at the edge of the cluster migrated with a significant increase in directionality compared to unconfined cells, which followed colony outgrowth, and a slight increase in speed (Fig. 1h–i). Similar results were obtained using glass coverslips manufactured to exhibit 50-µm-thick spacers (LifterSlipsTM), definitely ruling out that the pressure applied by the coverslip was accountable for cell migration (Supplementary Fig. 1e). To investigate whether differences of cell proliferation in confined versus unconfined environment could play a role in this process, we blocked cell proliferation by mitomycin, a DNA alkylating compound. Mitomycin treatment neither prevented nor increased directed migration to oxygen, demonstrating that proliferation was not involved in this process (Supplementary Fig. 2). Our observations of oxygen-directed migration were also reproduced using different cell lines, including HEK-293T, MCF12A, Hs578T, and HMECt, indicating that this property was not restricted to MCF10A and even not to mammary cells (Supplementary Fig. 3, Supplementary Movie 4). We have therefore established that eukaryotic cells in deep hypoxia had the ability to migrate directionally towards oxygen.

### Mitochondria are dispensable for oxygen sensing

**Fig. 2:**
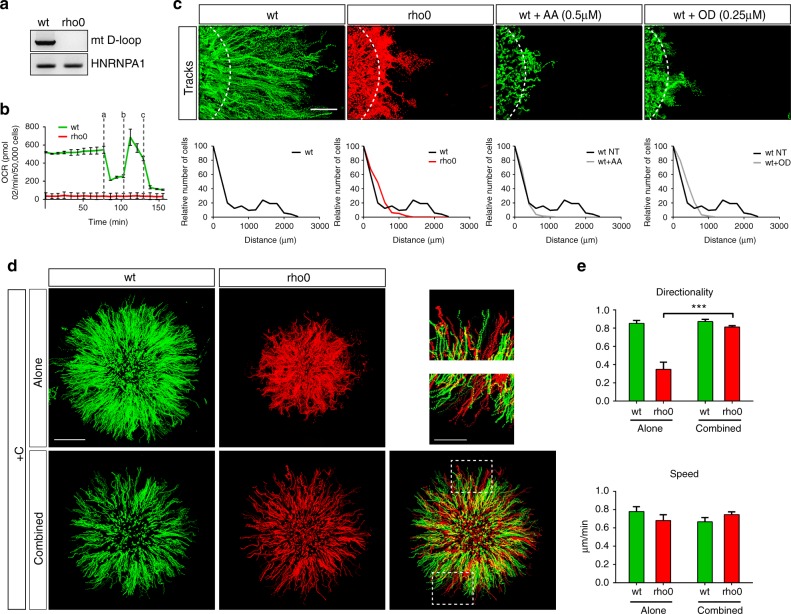
Mitochondria self-generate hypoxia but are nonessential for directed migration towards oxygen. **a** PCR amplification of mtDNA D-loop region and hnRNPA1 nuclear DNA in wt MCF10A and rho0 cells demonstrating the effective depletion of mtDNA from rho0 cells. **b** Oxygen consumption rate (OCR) of wt and rho0 cells before and following addition of (**a**) oligomycin D (0.5 µM), (**b**) FCCP (1 µM), (**c**) antimycin A 0.5 µM + rotenone (0.5 µM) (mean ± SD). This experiment demonstrates that rho0 cells are indeed unable to respire. **c** Tracking and redistribution of rho0 (red) and wt (green) cells set under confinement and treated or not with antimycin A (AA) or oligomycin D (OD) demonstrate that hypoxia generation is required for directed migration under confinement. Top panels: cell trajectories. White dotted-lines indicate the border of the cell cluster at 0 h. Bottom panels: relative distribution of MCF10A cells at the edge of the cluster at 0 h and 48 h post-confinement. Scale bar, 500 µm. **d** Tracking of confined rho0 (red) and wt cells (green) either alone or combined (1:1 ratio) for 24 h. Upper right: magnification of the framed regions. This experiment demonstrates that rho0 cells that do not respire are still capable of aerotactic migration provided that they are located within the oxygen gradient generated by wt cells. Scale bars; 1 mm (500 µm for magnification panels). **e** Mean values of directionality and speed from (**d**) (mean ± SD; *n* = 3 independent experiments). ***P < 0.001 by two-tailed Student’s *t*-test. +C confined

As the main oxygen consumer, we first investigated whether mitochondrial respiration was involved in oxygen sensing and oxygen-directed migration. To address this issue, we generated MCF10A cells devoid of functional mitochondria (rho0 cells, see Methods for details). Rho0 cells were fully depleted of mitochondrial DNA (Fig. 2a, Supplementary Fig. 4a), lacked cellular respiration (Fig. 2b), exhibited increased glycolysis (Supplementary Fig. 4b) and no longer activated HIF1A or its target genes under confinement, even though they maintained their capacity to upregulate HIF1A upon PHD inhibition (Supplementary Fig. 4c–d). Rho0 cells did not migrate directionally under confinement (Fig. 2c, Supplementary Movie 5), although their intrinsic motility in the classical wound healing assay or as isolated cells was not compromised (Supplementary Fig. 5). To confirm that the loss of directed motility resulted from their inability to self-generate hypoxia, we blocked mitochondrial respiration of wt MCF10A cells with two well-known inhibitors of the OXPHOS system, antimycin A (AA) and oligomycin D (OD). As seen previously with rho0 cells, these cells failed to activate the HIF1A pathway (Supplementary Fig. 4c–d) and to undergo directional migration under confinement (Fig. 2c), while their intrinsic motility was not significantly affected (Supplementary Fig. 5). However, when rho0 cells expressing H2B-mCherry and wt cells expressing H2B-GFP were mixed and confined, the former migrated similarly to wt cells with equivalent directionality and speed, indicating that the hypoxic conditions generated by wt cells drove directed rho0 cell migration (Fig. 2d–e, Supplementary Fig. 6, Supplementary Movie 5). Taken together, our findings show that mitochondrial respiration, the major oxygen consuming cellular process, was dispensable for directional migration towards oxygen and that it was only required for the self-generation of hypoxia.

### Aerotaxis is independent of the PHD/HIF pathway

**Fig. 3:**
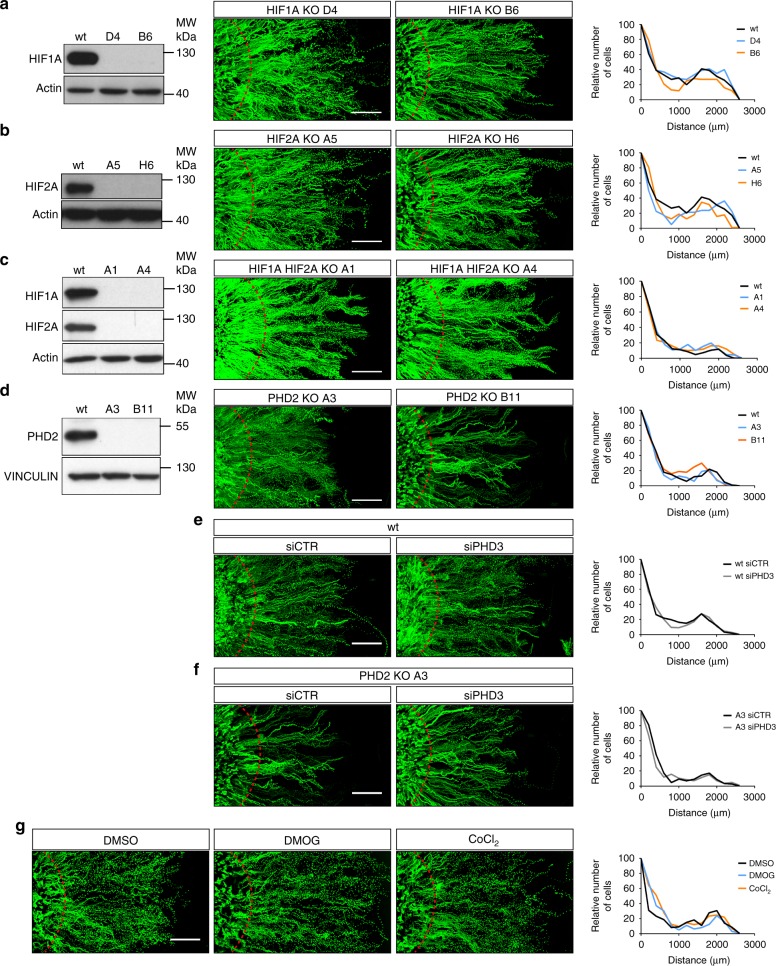
Oxygen chemotaxis is independent of the PHD/HIF pathway. **a**–**d** *HIF1A*, *HIF2A*, *HIF1A*+*HIF2A* and *PHD2* CRISPR/Cas9 KO clones characterisation, respectively, regarding O_2_-directed migration. Left panels: immunoblot validation of *HIF1A*, *HIF2A*, *HIF1A*+*HIF2A* and *PHD2* KO clones. To blot HIFs factors (**a**, **b**, **c**), cells were first pre-treated for 5 h with CoCl_2_ 300 µM before protein extraction, a condition that promotes HIF factors accumulation (cf. Supplementary Fig. 8f). Middle panels: cell trajectories under confinement. Red dashed lines indicate the border of the cell cluster at 0 h. Right panels: relative distribution of MCF10A KO clones versus wt cells at the edge of the cluster at 48 h. These experiments demonstrate that HIF factors deletion does not prevent aerotaxis. **e**, **f** Tracks and redistribution of wt and *PHD2* KO clone silenced for *PHD3* (siPHD3) or not (siCTR). **g** Tracks and redistribution of MCF10A cells treated with DMOG (50 µM) or CoCl_2_ (50 µM) to inhibit PHDs, or with vehicle only (DMSO). These experiments demonstrate that PHDs do not participate in O_2_-sensing during aerotaxis. Confinement was applied for 48 h (**a**–**g**). Scale bars, 500 µm

We then searched for mechanisms that could sense oxygen gradients at the cellular level and sustain directional steering towards oxygen. We first focused on the HIF pathway as it is the main cellular response to hypoxia and was shown above to be rapidly activated under confinement (Fig. 1d–e, Supplementary Fig. 1a–c). To investigate their roles in directed migration, *HIF1A* and *HIF2A* genes were invalidated in MCF10A cells using the CRISPR/Cas9 approach. *HIF1A* KO and *HIF2A* KO clones behaved as wt cells when confined (Fig. 3a–b, Supplementary Fig. 7). To rule out a possible redundancy between HIF1A and HIF2A, *HIF1A* was further inactivated in *HIF2A* KO clones. Again, double *HIF1A* and *HIF2A* knockout clones performed similarly to wt cells under confinement (Fig. 3c, Supplementary Fig. 7). These experiments demonstrated that HIF factors and possibly their targets were not involved in the process of oxygen chemotactism. However, PHDs, but not the HIF factors are the genuine oxygen sensors of the HIF pathway. Among the three PHDs known to date, PHD2 was the most abundant in MCF10A cells (Supplementary Fig. 8a). *PHD2* KO by CRISPR-Cas9 did not abolished directional mobility (Fig. 3d, Supplementary Fig. 8b). We also silenced *PHD3* since it was strongly expressed upon hypoxia (Supplementary Fig. 1c) or after *PHD2* invalidation despite the fact that it was poorly expressed under normoxic conditions (Supplementary Fig. 8c). However, *PHD3* silencing both in wt cells and *PHD2* KO cells did not affect directed migration under confinement (Fig. 3e–f, Supplementary Fig. 8d–e). Finally, to fully rule out a possible role of the PHD enzymes, we used two effective inhibitors of these enzymes, DMOG (dimethyloxalylglycine) and CoCl_2_. Although both DMOG and CoCl_2_ induced HIF1A stabilization, none of these inhibitors prevented the directed migration of cells under confinement, indicating that they were not involved in chemotaxis to oxygen (Fig. 3g, Supplementary Fig. 8f–g). Of note, in the absence of confinement, PHD inhibition by these compounds did not induce cells to break away from the cluster, indicating that the sole stabilisation of HIF factors was not sufficient to trigger directional migration (Supplementary Fig. 8h).

### Confinement generates ROS gradients

**Fig. 4:**
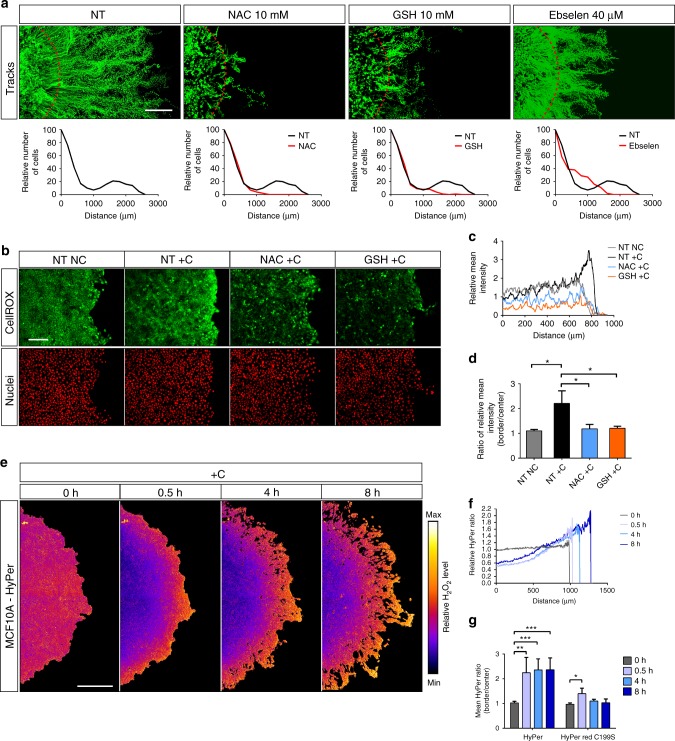
ROS are essential players of aerotaxis. **a** Tracking and redistribution of confined wt MCF10A cells upon treatment with antioxidants. NT non-treated, NAC N-acetyl-cysteine, GSH reduced glutathione; Ebselen. Top panels: cell trajectories over 48 h. Dashed red lines indicate the border of the cell clusters at 0 h. Bottom panels: relative distribution of MCF10A cells at the edge of the cluster at 48 h. These experiments demonstrate that antioxidants dampen aerotaxis. Scale bar, 500 µm. **b** Analysis of ROS accumulation with the chemical probe CellROX green unveils the ROS gradient generated under confinement (2 h): H2B-mCherry expressing MCF10A cells were confined (+C) or not (NC) and either not treated (NT) or treated with NAC (10 mM), GSH (10 mM) or Ebselen (40 µM). Images are representative of 3 independent experiments. Scale bar, 200 µm. **c** Mean CellROX fluorescence intensities (normalized to the NT NC condition) along the radius of the cell cluster were assessed from (**b**). **d** Border to centre ratios of CellROX green fluorescence intensity (mean ± SD; *n* = 3 independent experiments). These experiments demonstrate that ROS specifically accumulate at the edge of the cell cluster under confinement, a process that is abolished upon treatment by antioxidants. **e** Assessment of H_2_O_2_ concentrations with MCF10A cells expressing HyPer-3 under confinement. Relative H_2_O_2_ level is represented in pseudocolors (see Methods for details). Scale bar, 500 µm. **f** Relative H_2_O_2_ levels (normalized to the *t* = 0 h level) along the radius of the cell cluster computed from panel (**e**). **g** Mean border to centre ratios of HyPer-3 and HyPer-red-C199S signals calculated from panel (**e**) and Supplementary Fig. 10a (*n* = 4). ***P < 0.001, **P < 0.01, *P < 0.05 by two-tailed Student’s *t*-test. NC unconfined, +C confined

Oxygen is also a substrate for oxidative reactions supported by various oxidases, the activities of which result in the production of ROS. Although ROS can be detrimental to cells, they are also *bona fide* second messengers regulating a number of physiological processes^16,17^. To investigate the putative role of ROS in the transduction of oxygen sensing and directional migration, we first evaluated the effect of three known ROS inhibitors, N-acetylcysteine (NAC), reduced glutathione (GSH) and Ebselen. All these compounds severely impaired directional migration (Fig. 4a, Supplementary Fig. 9a), an effect that was not caused by the inhibition of intrinsic motility (Supplementary Fig. 9b–c), or the generation of hypoxia (Supplementary Fig. 9d), suggesting that ROS were involved in aerotaxis. Since oxygen forms a steep gradient at the periphery of the cell cluster (Fig. 1c), we wondered whether ROS production could be topologically dependent on oxygen concentrations. To analyse ROS distribution across the confined cell cluster, we used CellRox Green, a cumulative fluorescent probe of intracellular ROS. CellRox green labelling demonstrated a strong accumulation of ROS at the margin of the cluster, which was suppressed by NAC and GSH (Fig. 4b–d). To monitor ROS production in a more dynamic manner, we then expressed the fluorescent H_2_O_2_ sensor HyPer-3 in MCF10A cells together with its ROS insensitive mutant, HyPer red (C199S). Since both probes are sensitive to pH changes, co-expression of HyPer red (C199S) was performed to decipher which modification, redox or pH, was truly responsible for the observed changes in fluorescence (see Methods for further explanations). Although the HyPer-3 signal was uniform before confinement, a strong H_2_O_2_ gradient occurred at the margin of the cluster as early as 30 min after confinement (Fig. 4e–g, Supplementary Movie 6). A homogeneous decrease in HyPer red (C199S) fluorescence reflecting the already observed medium acidification under hypoxia (Supplementary Fig. 4b) was observed under confinement, but no increase in signal intensity could be detected along the radius of the cluster (Supplementary Fig. 10a). Therefore, the specific pattern obtained with the HyPer-3 probe could be definitely ascribed to a gradient of H_2_O_2_ production. Since mitochondria are the major source of cellular ROS as a by-product of cellular respiration, we then tested the mitochondria-targeted antioxidant MitoTEMPO, and S3QEL-2 (1-(3,4-Dimethylphenyl)-N,N-dipropyl-1H-pyrazolo[3,4-d]pyrimidin-4-amine), a cell-permeable selective suppressor of electron leak from the mitochondrial respiratory complex III. Both MitoTEMPO and S3QEL-2 were unable to profoundly impair directed migration under confinement (Supplementary Fig. 10b), confirming that mitochondria, even via ROS production, were not involved in aerotaxis. In addition, this result strongly suggested that the ROS involved in oxygen signalling were the products of one or several cytosolic or membrane-attached oxidases.

### EGFR steers cell migration towards oxygen

**Fig. 5:**
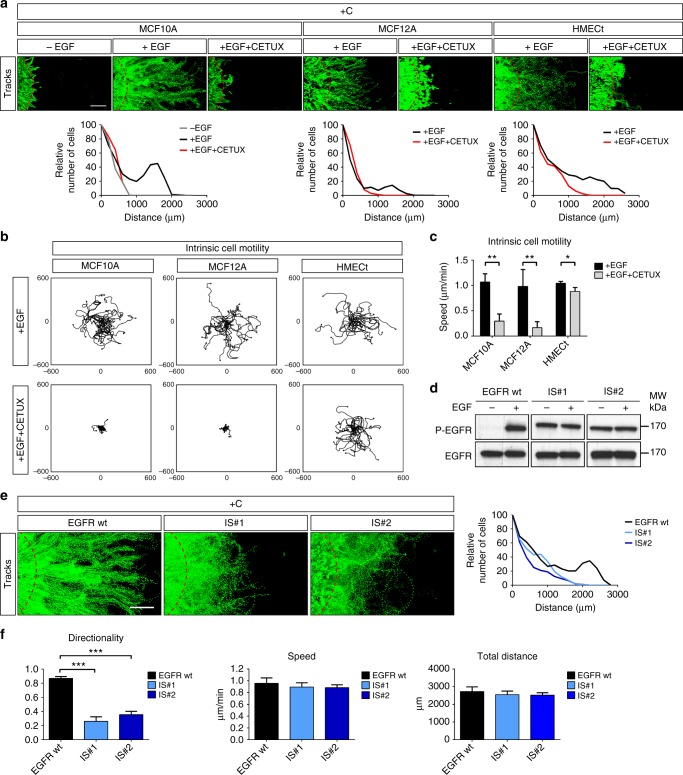
Aerotaxis of epithelial mammary cell depends upon EGFR activation. **a** Tracking and redistribution of confined MCF10A, MCF12A and HMECt cells, without EGF, with EGF or with EGF and the EGFR blocking antibody Cetuximab (CETUX). Top panels: representation of cell trajectories over 48 h of confinement. Red dotted-lines indicate the edge of the cell cluster at 0 h. Bottom panels: relative distribution at 48 h of MCF10A, MCF12A and HMECt cells treated or not with Cetuximab. EGF deficiency or Cetuximab abolished aerotaxis in all these cell types. Scale bar, 500 µm. **b** Individual cell tracking over a 12 h period of isolated MCF10A, MCF12A or HMECt cells seeded at 5% confluence with EGF and treated or not with Cetuximab (distances in µm). **c** Intrinsic cell motility (speed) assessed from experiments presented in (**b**) (mean ± SD; *n* = 3 independent experiments). These experiments (**b**–**c**) demonstrate that HMECt cells, unlike MCF10A and MCF12A, do not require EGF signalling to move. **d** Immunoblot of EGFR phosphorylation on tyrosine Y1173 showing the constitutive activation of EGFR in *IS#1* and *IS#2* clones knocked-out for endogenous EGFR and expressing the constitutively active EGFR mutant (D770_N771insNPG referred as INS). **e** Tracking and redistribution of confined wt EGFR expressing MCF10A cells and *IS#1* and *IS#2* clones in complete medium containing EGF. Left panels: representation of cell trajectories at 48 h of confinement. Red dashed lines indicate the border of the cell cluster at 0 h. Right panel: relative distribution of MCF10A cells expressing wt EGFR, and *IS#1* and *IS#2* clones at 48 h. Scale bar, 500 µm. **f** Mean values of directionality, speed and total distance calculated from (**e**) (mean ± SD; *n* = 3 independent experiments). These experiments demonstrate that *IS#1* and *IS#2* clones in which EGFR signalling is always turned-on are highly motile but miss directionality. +C confined. ***P < 0.001, **P < 0.01, *P < 0.05 by two-tailed Student’s *t*-test

Redox regulation was convincingly reported for a number of factors and enzymes involved in cell migration, including Rac1, RhoA, Ras, Src, Lyn, FAK, PDGFR and EGFR^18,19^. Since EGF is instrumental in MCF10A, MCF12A and HMECt cell growth and colony morphology, we focused on the potential role of EGFR in aerotaxis. Directed migration of MCF10A, MCF12A and HMECt cells under confinement was profoundly impaired in EGF-depleted medium or in medium supplemented with Cetuximab, a neutralising EGFR monoclonal antibody (Fig. 5a, Supplementary Fig. 11a). Interestingly, although Cetuximab severely reduced both MCF10A and MCF12A intrinsic motility in the isolated cell migration assay, it failed to profoundly affect HMECt cell behaviour in this assay (Fig. 5b, c, Supplementary Fig. 11b). This finding clearly dissociates oxygen chemotaxis from motility in the HMECt cell line and therefore argued in favour of a specific role for EGFR in steering epithelial cells towards oxygen. To further test this hypothesis, we expressed in wt MCF10A cells a constitutively active and EGF-independent EGFR mutant (D770_N771insNPG, further called INS mutant^20^). The endogenous *EGFR* gene was then knocked down in these cells by CRISPR/Cas9, leading to the creation of the MCF10A *IS#1* and *IS#2* clones (Fig. 5d). As expected, EGFR activation and cell growth, but also intrinsic motility of MCF10A IS*#1* and *IS#2*, became entirely independent of EGF (Fig. 5d, Supplementary Fig. 12a–c). However, MCF10A- *IS#1* and *IS#2* migration under confinement suffered from a dramatic loss of directionality but neither speed nor the total distance travelled were significantly affected (Fig. 5e, f, Supplementary Fig. 12d, Supplementary Movie 7). These observations strongly supported the idea that a precisely tuned EGFR activity was required for the directional steering towards oxygen.

### Confinement and redox regulation of EGFR

**Fig. 6:**
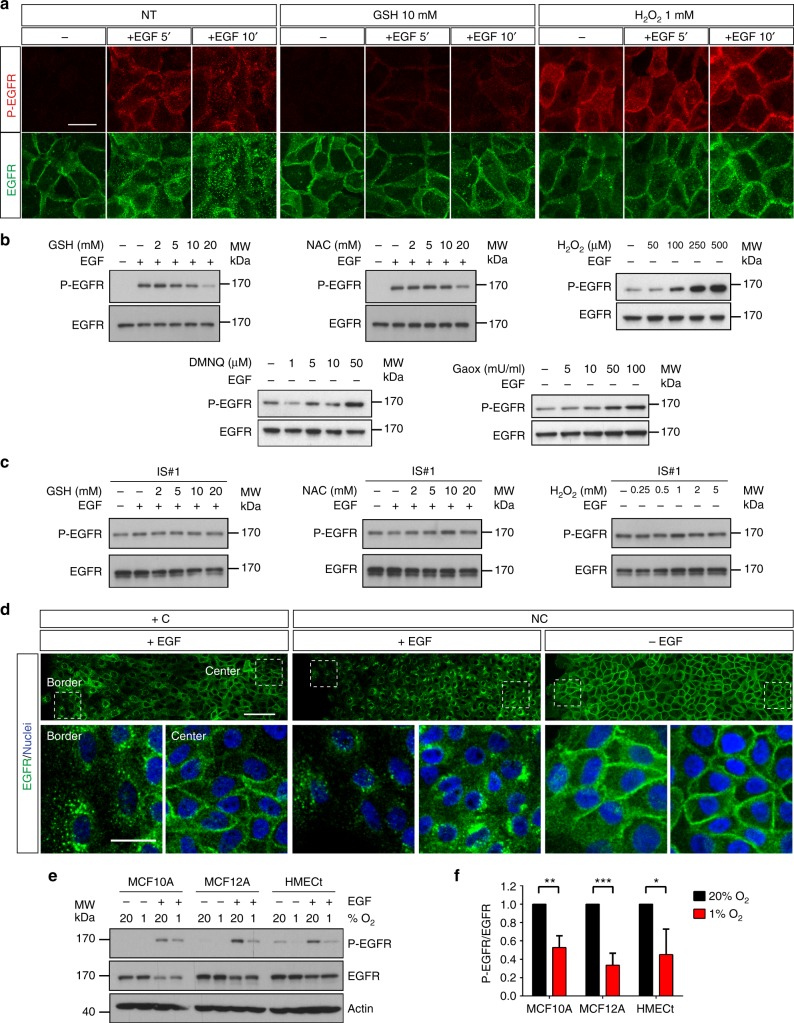
Confinement triggers a gradient of EGFR activity through ROS and hypoxia gradients. **a** Activation of EGFR was assessed by immunofluorescence, either by EGFR phosphorylation on tyrosine Y1173 (red), or total EGFR (green) internalisation upon treatment with reduced glutathione (GSH) or H_2_O_2_. EGF-starved MCF10A cells were pre-treated with either GSH (1 h) or H_2_O_2_ (10 min) before EGF (5 ng mL^−1^) was added for 5 or 10 min. Scale bar, 30 µm. **b** Immunoblot analysis of EGFR phosphorylation (P-Y1173) upon increasing concentrations of GSH, NAC, H_2_O_2_, DMNQ (an endogenous generator of H_2_O_2_) or galactose oxidase (GAOX), an enzymatic system generating low amount of H_2_O_2_ in a continuous manner. These experiments confirm that EGFR activation is promoted by oxidant and inhibited by antioxidants. **c** Immunoblots analysis of mutant-EGFR phosphorylation in *IS#1* clone upon treatment with GSH, NAC and H_2_O_2_. These experiments demonstrate that the constitutively activated EGFR (INS) mutant is insensitive to redox regulation. (**d**) EGFR activation assessed by EGFR internalisation (green) across the cell cluster 6 h after confinement (+C) or unconfined (NC) in normal medium (+EGF) or in medium without EGF (−EGF) (representative of 3 independent experiments). Top panels: macroscopic view of the left side of the cell clusters. Bottom panels: magnification of the framed regions. Confinement induces a differential activation of EGFR between the centre and the border of the cell cluster. Scale bars, 100 µm (top), 30 µm (bottom). **e** EGFR phosphorylation (Y1173) in MCF10A, MCF12A and HMECt cells treated or not with EGF under normoxic or hypoxic (1% O_2_ for 6 h) conditions. **f** Relative activation of EGFR (ratio of phospho-EGFR (Y1173) over total EGFR) by EGF in MCF10A, MCF12A and HMECt cells under 20% or 1% O_2_ calculated from (**e**) (mean ± SD; *n* = 3 independent experiments). Hypoxic conditions dampen EGFR activation. NC unconfined, +C confined. ***P < 0.001, **P < 0.01, *P < 0.05 by two-tailed Student’s *t*-test

**Fig. 7:**
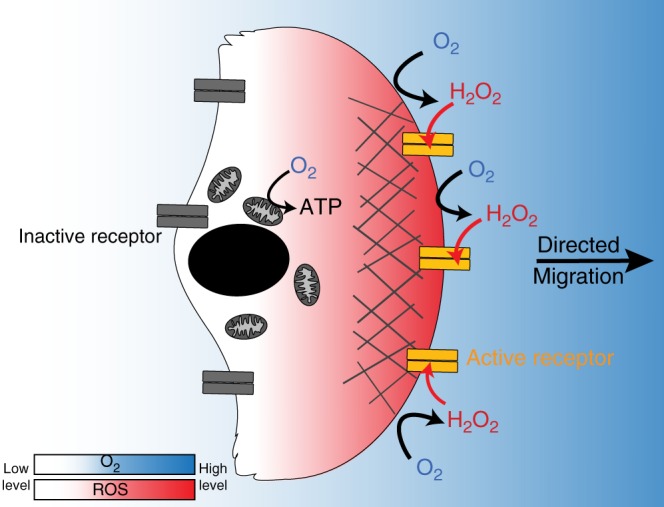
Model of directed migration of mammary cells in oxygen gradients. Mitochondrial respiration generates hypoxia and an oxygen gradient that in turn generates a ROS gradient since oxygen is required for ROS production. At the cell level, these ROS, that are likely more abundant at the front edge than at the lagging edge induce a differential activation of EGF receptors that sustains directionality

Lastly, ROS dependent regulation of EGFR activation was thoroughly analysed at the level of the cell cluster. Following EGF binding, this receptor undergoes phosphorylation, internalisation in clathrin-coated vesicles and downstream degradation. The use of EGFR- or phospho-EGFR-specific antibodies confirmed that EGF stimulation triggered EGFR phosphorylation and endocytosis as demonstrated by immunofluorescence (Fig. 6a left panel). This process is dampened upon GSH or NAC treatment in several cell types (Fig. 6a, b, Supplementary Fig. 13). Conversely, oxidative stress, by incubation with H_2_O_2_, or continuous generation of low amount of exogenous H_2_O_2_ by the Gaox (galactose/galactose oxidase) system or endogenous generation of H_2_O_2_ by DMNQ (2,3-Dimethoxy-1,4-naphthoquinone) strongly potentiated the activation of EGFR, which corroborated the previously reported redox regulation of this receptor^21,22^ (Fig. 6a, b for MCF10A cells, Supplementary Fig. 13 for other cell types). In contrast, the level of activation of the EGFR INS mutant was entirely ROS-independent, being neither dampened by GSH or N-acetylcystein, nor increased by hydrogen peroxide, even at high concentrations (Fig. 6c). This lack of redox regulation of the constitutively active mutant might account for the already shown high mobility but absence of directionality of *IS#1* and *IS#2* cells (Fig. 5e, f). Furthermore, we observed that under confinement, receptor internalization and therefore EGFR activation was restricted to the periphery of the MCF10A cell cluster (Fig. 6d, left panel, Supplementary Fig. 14 for MCF12A and HMECt cells), a situation clearly reminiscent of the previously observed ROS gradient topology (Fig. 4b–g). In contrast, in the absence of confinement, a uniform activation of EGFR across the cluster was observed in EGF containing medium (Fig. 6d central panel, Supplementary Fig. 14 for other cell types), in agreement with the unvarying ROS concentrations previously measured under these conditions (Fig. 4b–g). As expected, in the absence of EGF, no EGFR activation was observed (Fig. 6d, right panel). Of note, the generation of the EGFR activation gradient in the cell cluster was clearly dependent on the capacity of cells to generate hypoxia under confinement. Indeed, Rho0 cells or MCF10A cells treated by antimycin A or oligomycin D showed a strong and uniform EGFR activation across the cluster (Supplementary Fig. 15). To further confirm the relationship between EGFR activation by its ligand and oxygen levels, EGFR activation was compared under normoxic and 1% hypoxic conditions. EGF-dependent EGFR phosphorylation in MCF10A, MCF12A and HMECt cell lines was significantly reduced in hypoxia compared to normoxia and therefore relied on oxygen availability (Fig. 6e, f). Taken together, these observations strongly suggest that cells located at the periphery of the cluster and exposed to steep oxygen and ROS gradients undertake directional migration through the front/tail differential activation of the EGFR receptor. The schema of the proposed mechanism is shown in Fig. 7.

## Discussion

Altogether, our results indicate that the local production of ROS at the periphery of confined cell clusters, itself dependent on oxygen availability, may potentiate EGFR activation, whereas hypoxia at the centre of clusters contributes to its inhibition as previously reported^23^. Due to the steep gradients, this phenomenon is probably similar at the cellular level, leading to a stronger EGFR activation at cell surfaces facing higher oxygen concentrations. An imbalanced receptor activation could therefore explain directional cell migration by promoting more efficient lamellipodia nucleation and stabilisation at the edge of cells confronted to increased ROS concentrations (Supplementary Movie 8). Continuous oxygen consumption by migrating cells would perpetuate both oxygen and ROS gradients and therefore preserve directional migration along the same radial axis.

Of note, the unique experimental system that we used, relying on cell respiration itself to generate hypoxia, clearly mimics physiological and pathological situations in which hypoxia and steep oxygen gradients result from unbalanced oxygen transport and consumption rates. The level of hypoxia at the centre of the cell cluster was <1%, well below the oxygen concentration required to activate the hypoxia response pathway. In addition, the oxygen gradient at the edge of the cell cluster, with an oxygen concentration ranging from 10 to 1% in <100 µm, was similar to that described at the periphery of a blood capillary^13,24^. More importantly, the gradient was steep enough to allow a clear difference in oxygen concentration between the leading and the lagging edges of migrating cells.

Mitochondrial respiration was not necessary for aerotaxis, suggesting that this process was not a form of energy taxis. Furthermore, even though the major hypoxia response through HIF/PHD pathway was activated, it did not support directed migration. Instead, we identified the receptor tyrosine kinase (RTK) EGFR for mammary MCF10A, MCF12A and HMECt cells, as a potential redox sensor to mediate directed migration towards higher oxygen levels. A cysteine (C797) located in the hinge region of the ATP binding site of EGFR was recently identified as a target of redox regulation^21,22^. Interestingly, a rise in H_2_O_2_ production was observed upon EGF binding^25–28^, which depended on a calcium-dependent H_2_O_2_ production by the NADPH oxidase Duox1 in both airway and epidermal epithelial cells^28^. In addition to the fact that redox modifications of critical thiols by H_2_O_2_ could directly activate EGFR, it has been known for a long time that H_2_O_2_ simultaneously inhibits protein tyrosine phosphatases (PTP) involved in signal inactivation^29^. Therefore, it is reasonable to presume that the H_2_O_2_ gradient detected under confinement in our experiment generates a differential and sustained EGFR activation from the leading to the lagging edges of the cell cluster, resulting in lamellipodia stabilisation at the surface most exposed to ROS and consequently directional migration towards oxygen. How this differential activation of EGFR in mammary cells is converted into cell polarization and cell movement will require additional work^30–33^. Of note, human renal embryonic epithelial HEK-293T cells are well-known as not expressing EGFR^34^. It is therefore likely that aerotaxis can be mediated by proteins other than EGFR in cell types from other tissues.

## Methods

### Drugs and inhibitors

The following drugs and inhibitors; NAC, GSH, DMOG, CoCl_2_, antimycin A, FCCP (carbonyl cyanide 4 (trifluoromethoxy)phenylhydrazone), D-galactose, GAOX (galactose oxidase), puromycin and mitomycin were purchased from Sigma-Aldrich (USA). Oligomycin D was ordered from Enzo life sciences (USA) and cetuximab (Erbitux) was obtained from Merck Biopharma (Germany). S3QEL-2, DMNQ and Ebselen (2-Phenyl-1,2-benzisoselenazol-3(2H)-one) were purchased from Bertin Pharma (France).

### Cell lines and cell culture

The mammary epithelial cell lines MCF10A and MCF12A, two spontaneous immortalised but non-transformed cell lines were obtained from the American Type Culture Collection. These cells were grown in DMEM/F12 medium supplemented with 5% horse serum, EGF (10 ng mL^−1^), insulin (10 µg mL^−1^), cholera toxin (100 ng mL^−1^), hydrocortisone (0.5 µg mL^−1^) and penicillin plus streptomycin. Hs578T and 293T cells, gifts from A. Puisieux and F-L Cosset, respectively, were cultured in DMEM medium supplemented with 10% foetal calf serum. HMECt are immortalised human mammary epithelial cells infected with a retrovirus carrying hTERT^35^. HMECt, kindly provided by R. A. Weinberg, were grown in DMEM/F12 medium supplemented with 10% foetal bovine serum, EGF (5 ng mL^−1^), insulin (10 µg mL^−1^), hydrocortisone (0.5 µg mL^−1^) and penicillin plus streptomycin. When indicated, fluorescently labelled cell populations were generated by infection with lentiviral vectors expressing either H2B-GFP or H2B-mCherry and sorted by flow cytometry. All the cell lines used during this work were tested for *Mycoplasma* contamination by the MycoAlert Mycoplasma Detection Assay (Lonza) and were negative.

### Generation of the MCF10A rho0 cell line

Rho0 were generated by growing MCF10A cells for 8 weeks in complete MCF10A medium supplemented with 50 ng mL^−1^ ethidium bromide, 4.5 g L^−1^ D-glucose, 50 µg mL^−1^ uridine and 1 mM pyruvate as previously described^36^. Removal of mtDNA was controlled by qualitative PCR performed on total DNA using specific primers for D-Loop (MitoDNA-F: 5′-acccagacaattataccctagc-3′ and MitoDNA-R: 5′-gagcccgtctaaacattttcaatg-3′) and Histone H1 DNA (Histone H1-F: 5′-atgagctcatgaccgagaattccacgtccg-3′ and Histone H1-R: 5′-atcccgggcaaacttcttcttgcc-3′)^37^. Rho0 cells were then maintained in complete MCF10A medium supplemented with 4.5 g L^−1^ D-glucose, 50 µg mL^−1^ uridine and 1 mM pyruvate.

### Gene silencing with siRNA of *PHD3*

Transfection with siRNA (10 nM) was performed in antibiotic-free medium with the transfection reagent RNAi-MAX (Invitrogen, Thermofisher). SiRNAs including the siRNA negative control were purchased from IDT DNA Technologies. Sequence of the sense strand of *PHD3* siRNA is as follows: siPHD3 5′-guaauacuaucaagagaagagccta-3′.

### Real-time RT-qPCR analysis of mRNA expression and primers

Total RNAs were extracted using Tri Reagent (Sigma-Aldrich, USA) according to the manufacturer’s recommendations. RNAs were then reverse-transcribed with SuperScript II reverse transcriptase (ThermoFisher, USA) following the manufacturer’s instructions and analysed by RT-qPCR and SYBR green on a Step One apparatus (Applied Bioscience/ThermoFisher, USA). PCR primers used in RT-qPCR experiments were purchased from Integrated DNA technologies (USA). CAIX-F: 5′-cttcctcagcgatttcttcca-3′, CAIX-R: 5′-ccttttgccagagttgacgag-3′; PHD3-F: 5′-cctgttccatttcccggata-3′, PHD3-R: 5′-ttcctcctgtccctcatcg-3′; GLUT-1-F: 5′-ggccacaaagccaaagatg-3′, GLUT-1-R: 5′-gtgccatactcatgaccatcg-3′; VEGFA-F: 5′-gcgctgatagacatccatga-3′, VEGFA-R: 5′-ccatgaactttctgctgtcttg-3′; CXCR4-F: 5′-gtacttgtccgtcatgcttct-3′, CXCR4-R: 5′-aaatcttcctgcccaccatc-3′; PAI-1-F: 5′-tcttccacaaatcagacggca-3′, PAI-1-R: 5′-agcaatgaacatgctgagggtgtc-3′; MMP9-F: 5′-cgtcgaaatgggcgtct-3′, MMP9-R: 5′-acatcgtcatccagtttggtg-3′; ZEB2-F: 5′-gatcagatggcagttcgcat-3′, ZEB2-R: 5′-cctttttctcccccacactt-3′; LOX-F: 5′-ttcccacttcagaacaccag-3′, LOX-R: 5′-acattcgctacacaggacatc-3′; PHD1-F: 5′-ctaagggcttgggaaggg-3′, PHD1-R: 5′-gcctactgcgctcagaa-3′; PHD2-F: 5′-gtcacacatcttccatctccat-3′, PHD2-R: 5′-ggcagctacaaaatcaatggc-3′. Data were presented as mean ± S.D.

### Retroviral vectors

Retroviral vectors expressing wild type human EGFR (Addgene plasmid#11011) or EGFR mutant D770_N771insNPG (Addgene plasmid #11016), further mentioned as INS mutant, were gifts from Heidi Greulich^20^. The lentivector expressing H2B-EGFP was constructed by cloning the H2B-EGFP sequence from LV-GFP (a gift from Elaine Fuchs, Addgene plasmid #25999) into CSII-EF-MCS vector. The lentivector expressing H2B-mCherry was constructed by cloning the H2B sequence from LV-GFP into the CSII-EF-mCherry vector. CSII-HyPer and CSII-HyPer-C199S were constructed by cloning the HyPer and HyPer-C199S coding sequences from pCI-HyPer3 and pCI-HyPer-Red-C199S, respectively (gifts from Vsevolod Belousov, Addgene plasmids #42131 and #48252), into CSII-EF-MCS^38,39^. The lentivector expressing Lifeact-mCherry was constructed by cloning the Lifeact sequence from Addgene plasmid # 54491 into the CSII-EF-mCherry vector.

### Construction of CRISPR/Cas9 knockout clones

To knockout *HIF*, *PHD* and *EGFR* genes in MCF10A, we used the LentiCRISPR V2 plasmid (a gift from Feng Zhang, Addgene plasmid #52961). Oligonucleotides pairs were hybridized and cloned into the LentiCRISPR V2 vector linearized with BsmB1 to generate the following MCF10A clones^40^. *HIF1A* KO clones B4 and B6 (HIF1AGuide#7S: 5′-caccgaagtgtaccctaactagccg-3′, HIF1AGuide#7AS: 5′-aaaccggctagttagggtacacttc-3′), *HIF2A* KO clone A5 (HIF2AGuide#1S: 5′-caccgcaaggcctccatcatgcgac-3′, HIF2AGuide#1AS: 5′-aaacgtcgcatgatggaggccttgc-3′), *HIF2A* KO clone H6 (HIF2AGuide#2S: 5′-caccggctgattgccagtcgcatga-3′, HIF2AGuide#2AS: 5′-aaactcatgcgactggcaatcagcc-3′), *PHD2* KO clone A3 (PHD2Guide#2S: 5′-caccggtgggcccacaccagcattc-3′, PHD2Guide#2AS: 5′-aaacgaatgctggtgtgggcccacc-3′), MCF10A EGFR KO clones *IS#1* and *IS#2* (EGFR5′Guide#5S: 5′-caccgttactcgtgccttggcaaac-3′, EGFR5′Guide#5AS: 5′-aaacgtttgccaaggcacgagtaac-3′). To generate *HIF1A/HIF2A* double knockout clones A1 and A4, *HIF2A* KO clone H6 was further knocked-out for *HIF1A*.

Lentiviruses were produced in 293T cells by transfection of LentiCRISPR V2 plasmids together with pMD2.G and psPAX2 helper plasmids (a gift from Didier Trono, Addgene plasmids #12259 and #12260) following Addgene′s instructions. To generate knockout clones, MCF10A cells were infected at a multiplicity of infection of one or less with the corresponding LentiCRISPR V2 viruses and selected with puromycin (1 µg mL^−1^) for 5 days. Cells were then cloned in 96-well plates by limiting dilution. Isolated clones were characterized by immunoblotting and RT-qPCR analysis, and validated by sequencing. For each gene knockout, two different clones were selected and further tested for migration.

### Immunoblotting

Immunoblots were performed following standard procedures. Cell lysates prepared using RIPA buffer (50 mM Tris-HCl pH 7.5, NaCl 150 mM, NP40 1%, sodium deoxycholate 0.25%, EDTA 1 mM, and proteases and phosphatases inhibitor cocktails) were separated in 10% SDS-PAGE gels, transferred to PVDF membranes using the Trans-Blot Turbo System (Bio-Rad, USA) and probed with the following primary antibodies: HIF1A (BD Bioscience #610958; dilution: 1/300), HIF2A (Novus Bio #NB100-122; dilution: 1/300), PHD2 (Novus Bio #NB100-137; dilution: 1/500), EGFR (Cell Signaling #4267S; dilution: 1/1000), Phospho-EGFR (anti-Tyr1173 Santa Cruz #sc-12351; dilution: 1/500), Vinculin (Santa Cruz #sc-55465; dilution: 1/2000), Actin (Covalab #90010; dilution: 1/5000). Primary antibodies were detected with either anti-rabbit-, goat- or mouse-specific secondary antibodies conjugated with horseradish peroxidase (HRP, Dako) and detected by chemiluminescence (Roche Life Science, Germany). For the immunoblots depicted in Fig. 6b,c and Supplementary Fig. 13, cells were first starved for 18 h in EGF-free medium before they were treated with H_2_O_2_ for 5 min, DMNQ for 15 min or GAOX (indicated amounts) and D-galactose (3 mM) for 20 min. For antioxidant treatments, cells were starved in EGF-free medium and subsequently treated for 1 h with NAC or GSH in EGF-free medium and finally incubated with EGF (10 ng mL^−1^) for 5 min. For HIF2A blotting, nuclear lysates were prepared instead of total lysates to avoid migration of a non-specific band at the same molecular weight. Briefly, cells were collected by centrifugation. Cell pellets were gently resuspended in an hypotonic buffer (20 mM Tris-HCl pH 7.4, 10 mM NaCl, 3 mM MgCl_2_) and incubated on ice for 15 min. NP40 (0.5% vol/vol) was subsequently added and nuclei were pelleted by centrifugation at 3000 rpm at 4 °C for 10 min. Pellets were finally resuspended in RIPA buffer and incubated on ice for 30 min. Uncropped western-blots are displayed in Supplementary Figure 16.

### Immunofluorescence analysis

For immunofluorescence imaging, cells grown on glass coverslips (or IBIDI µ-dish 35 mm) were fixed in 4% paraformaldehyde, washed three times with PBS, permeabilised with 0.5% Triton X-100 in PBS for 10 min and washed again three times with PBS. Coverslips were then blocked with 3% BSA in PBS for 1 h, incubated with antibodies against HIF1A (BD Biosciences #610958; dilution: 1/100), EGFR (Santa Cruz #sc-101; dilution: 1/100) and Phospho-EGFR (anti-Tyr1173 Santa Cruz #sc-12351, dilution: 1/50), washed, stained with fluorescent secondary antibodies, washed, counterstained with Hoescht and finally mounted with fluorescent mounting medium (Dako) under a glass coverslip. Images were acquired with a Zeiss LSM 780 NLO laser confocal microscope (Zeiss, Germany). For EGFR and Phospho-EGFR imaging, three stacks of 2 µm were acquired and compiled into a single image by projection of the max intensity of each stack.

### Imaging of mitochondria

MCF10A wild type and rho0 cells were stained using the MitoTracker (MitoTracker^TM^ Deep Red FM, Life Technologies). Briefly, cells seeded onto coverslips were treated 20 min at 37 °C with MitoTracker (0.2 µM) in complete medium. Cells were then washed and incubated for 5 min in complete medium supplemented with 1 µM SYBR green for mitochondrial and nuclear DNA staining. Live cells were imaged with an inverted Zeiss LSM 780 NLO laser confocal microscope (Zeiss, Germany).

### Observation of cell migration under hypoxic confinement

To study cell migration in oxygen gradients, we developed the following experimental approach. Four thousand cells expressing H2B-GFP or H2B-mCherry (MCF10A, HMECt, MCF12A, 293T or Hs578T) resuspended in 2 µL of medium were plated as a small drop at the centre of each well in 24-well culture plates (Falcon/Corning) or ImageLock plates (Essen BioScience). Cells were then incubated for 4 h at 37 °C in a humidified chamber to adhere to the culture dish, before 1 mL of complete medium was added to the wells. After 24 h, 14 mm in diameter glass coverslips (Waldemar Knittel, GmbH, Germany) were immersed in the medium to cover and confine the cell cluster. Mitochondrial respiration under confinement rapidly generates hypoxia and hypoxic gradients and triggers directed cell migration towards higher oxygen tension. Live-cell migration under confinement was imaged and analysed for 24 h, 48 h or 72 h with the live-cell analysis Incucyte ZOOM system (Essen BioScience, USA). Stacks of images were aligned when necessary with the StackReg ImageJ plugin. Representative bright field pictures taken at 0 and 48 h were presented together with images representing cell tracks. The latter were generated by projection of time-lapse image series (30 min interval for 48 h) of fluorescently labelled cell nuclei, using the Z-project ImageJ plugin. To visualize and compare the redistribution of cells after migration under confinement, fluorescent time-lapse images were processed with the following ImageJ protocol; Find maxima-List-Results-Distribution. The results from three experiments were represented as curves, providing the mean fraction of cells (in %) detected per successive 200 µm wide distances, relative to the number of cells spread over the first 0–200 µm distance.

### Cell migration analysis and cell tracking

Clusters of nuclei-labelled cells plated on 24-well ImageLock plates (Essen BioScience) were subjected or not to confinement and imaged every 30 min for various periods of time. Nuclei displacements were tracked manually in three independent experiments consisting of more than 20 cells per condition representative of the entire margins of the cell cluster using the ImageJ software. Trajectories were represented as XY graphs. Cell migration parameters were calculated with an in-house-designed Excel algorithm (Microsoft). Displacement: shortest distance between the initial and final position of a given cell. Total distance: sum of distances travelled for 30 min time periods over 24 or 48 h. Directionality: displacement divided by total travelling distance. Speed: total travelling distance divided by time. Intrinsic cell motility was analysed similarly except that cells were seeded at 5% confluence in 24- or 96-well plates.

For the scratch wound assay, 65,000 cells plated on 96-well ImageLock plates (Essen BioScience) were left to adhere for 5 h at 37 °C and then scratched (800 µm width) with the Wound Maker (Essen BioScience). Tested compounds and corresponding controls were added just after scratching, and wound closure was followed and evaluated with the Incucyte Live-Cell Imaging System and dedicated software (Essen Bioscience). Cell migration was evaluated as a function of the wound closure efficiency when controls reached 100% wound repair. Results presented as graphs indicated the mean relative wound confluence in percent extrapolated from three independent experiments, each one performed in triplicate.

### Visisens evaluation of local hypoxia

Hypoxia under confinement was monitored using the VisiSens detector unit DUO1, the oxygen sensor foil SF-RPSu4 and the associated AnalytiCal1 software (PreSens, Germany). MCF10A cells were spotted in a 12-well plate as described earlier and a glass coverslip pre-coated by PreSens with the proprietary oxygen sensor (equivalent to SF-RPSu4 sensor foil) was used to confine cell clusters. Local oxygen tension close to the cells was then measured for 1 h at 5 min intervals by placing the detector unit under the 12-well plate facing the sensor. Visisens calibration was performed by exposing the SF-RPSu4 sensor foil to ambient air (21% oxygen) or to a saturating Na_2_SO_3_ solution for 10 min (0% oxygen). Unfortunately, this setup did not allow the direct visualisation of the cells under microscopy, the foil being opaque. Conversely, it was not possible to grow and migrate cells onto the sensor foil so that oxygen levels might be monitored by the specific camera from below, while simultaneously observing cells from the top.

### Measurement of OCR and ECAR

OCR (oxygen consumption rate) and ECAR (extracellular acidification rate) were measured using the Seahorse Bioscience XF24 Extracellular Flux Analyzer (Seahorse Bioscience, North Billerica, MA) according to the manufacturer’s protocol. Cells were seeded in normal growth medium 24 h before measurement at a density of 5 × 10^4^ cells/well. The assay was performed in XF minimal basal medium (Seahorse Bioscience) supplemented with 25 mM glucose, 1 mM sodium pyruvate, 4 mM glutamine, 10% FBS at pH 7.4. Before measurement, cells were pre-incubated in this medium for 1 h at 37 °C in a humidified atmosphere without CO_2_. Oligomycin D (0.5 µM), FCCP (1 µM), rotenone (0.5 µM), antimycin A (0.5 µM) were used to evaluate mitochondrial respiratory capacity and glycolytic capacity. The data were normalized with the number of cells in each well and presented as the mean of five different wells in two independent experiments.

### ROS detection in situ with CellROX Green

To analyse oxidative stress, cells were incubated with the indicated drugs for 2 h before CellROX Green reagent (Molecular Probes, ThermoFisher) was added to the medium at a final concentration of 5 μM for 30 min at 37 °C. Cells were then washed three times with PBS and placed under confinement for 2 h. Cells were finally fixed for 20 min in 4% paraformaldehyde and washed twice in PBS. CellROX Green fluorescence was then measured (Ex. 488/Em. 520) by confocal microscopy using identical settings (Zeiss LSM 780 NLO, Zeiss, Germany). Experiments were performed in triplicate.

### Detection of hydrogen peroxide with the fluorescent biosensor HyPer-3

To monitor cytosolic hydrogen peroxide concentration and to assess intracellular pH variations, MCF10A cells were co-infected with HyPer-3 and HyPer-Red C199S expressing lentiviruses^38,39^. The HyPer-3 protein is a circular permuted yellow fluorescent protein (cpYFP) inserted into the regulatory domain of the prokaryotic H_2_O_2_-sensing protein, OxyR. Cysteine 199 oxidation to a sulphenic acid by H_2_O_2_ results in structural modification of OxyR-RD, which alters the fluorescence excitation spectrum of YFP. As a protein itself, HyPer-3 is particularly relevant as an indicator of cellular protein oxidation. Of note, HyPer-3 demonstrates submicromolar affinity to hydrogen peroxide and is insensitive to other oxidants, such as superoxide, oxidized glutathione, nitric oxide and peroxinitrite. HyPer-3 does not cause artefactual ROS generation upon light exposition and can be used for detection of rapid changes in H_2_O_2_ concentration under various physiological and pathological conditions. However, HyPer-3 remains sensitive to pH changes. Therefore, HyPer-Red-C199S, a red version of the HyPer-3 probe with an additional C199 mutation that renders the probe insensitive to H_2_O_2_ but still sensitive to pH changes was co-expressed with HyPer-3. Live-imaging was performed at 37 °C and in 5% CO_2_ with a Zeiss LSM 780 (10X objective). To evaluate H_2_O_2_ concentrations in cellulo, two fluorescence measurements were carried out at 525/30 nm, excitation being set either at 488 nm or 405 nm. HyPer-3 oxidation was then assessed by calculating the 488/405 ratio indicative of the intracellular H_2_O_2_ concentration. For the evaluation of pH changes, fluorescence detection of the HyPer-Red-C199S sensor was carried out in a single 647/75 nm channel with excitation at 560/40 nm. Image analysis was performed with ImageJ. For HyPer-3 imaging, pictures were first processed to eliminate background before the 488/405 ratio was calculated. Resulting images were displayed in pseudocolors. The HyPer-3 ratio and the HyPer-Red C199S fluorescence intensity were quantified and represented along the *X*-axis using the ImageJ plugin “plot profile”.

### Quantification and statistical analysis

Statistical details of experiments can be found in the Figure legends. Data are usually presented as mean ± SD. Statistical significance was determined using a two-tailed Student’s *t*-test (*n* = 3; replicated 3 times) with the following conventions: *P *<* 0.05, **P *<* 0.01, ***P *<* 0.001.

## Electronic supplementary material

Supplementary Information

Description of Additional Supplementary Files

Supplementary Movie 1

Supplementary Movie 2

Supplementary Movie 3

Supplementary Movie 4

Supplementary Movie 5

Supplementary Movie 6

Supplementary Movie 7

Supplementary Movie 8

## Data Availability

All relevant data are available from the authors. Data sharing is not applicable to this work as no datasets were generated or analysed during the current study.
