# Supplementary material for: Redox regulation of EGFR steers migration of hypoxic mammary cells towards oxygen
Source: Nat Commun. 2018 Oct 31;9:4545. doi: 10.1038/s41467-018-06988-3 (PMC6208388; doi:10.1038/s41467-018-06988-3)

**Supplementary Figure 1**

| **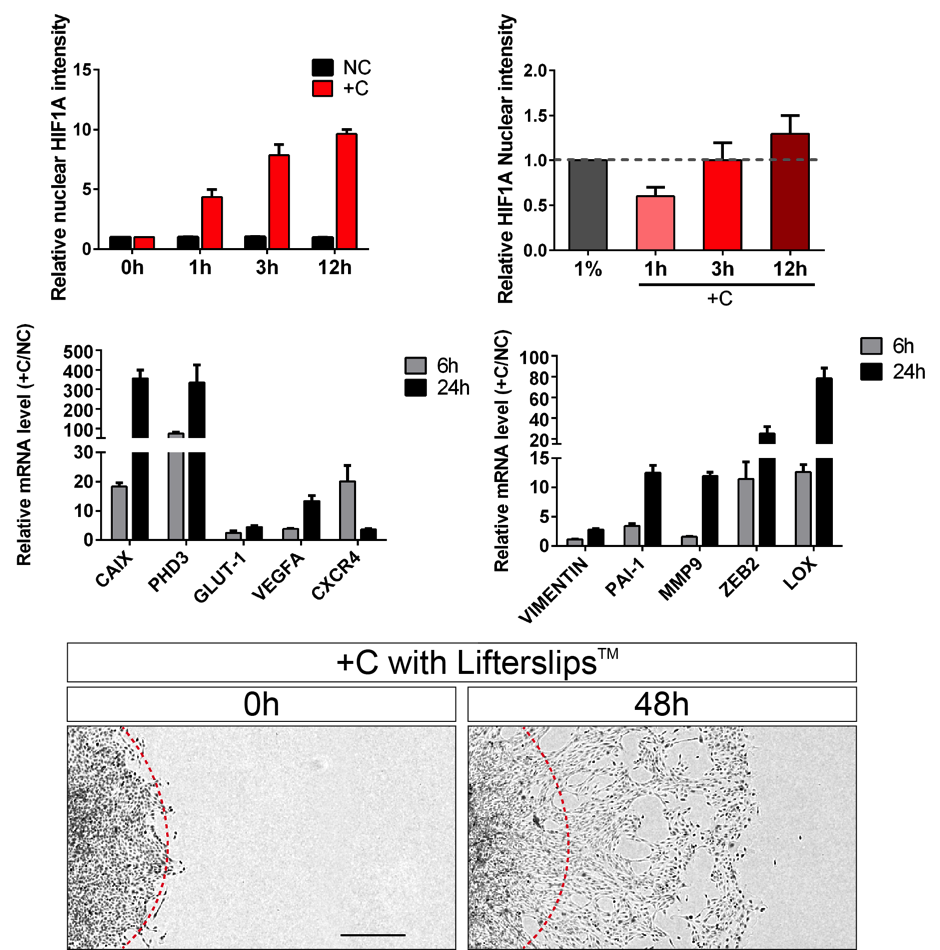**  **a**  **c**  **e**  **d**  **b** |
| --- |

**Induction of EMT and HIF target genes under confinement**

**e**

**(a)** Relative mean intensity of nuclear HIF1A in the whole cluster after confinement for 0 h, 1 h, 3 h and 12 h (mean ± SD; n = 3 independent experiments). **(b)** Relative mean intensity of nuclear HIF1A in the whole cluster after confinement for 1 h, 3 h and 12 h compared to nuclear HIF1A accumulation in MCF10A cells grown in 1% hypoxia for 12 h (mean ± SD; n = 3 independent experiments). **(c)** Relative expression of HIF1A target genes in MCF10A cells, confined (for 6 h and 24 h) versus unconfined, measured by RT-qPCR (mean ± SD; n = 3 independent experiments). **(d)** Relative expression of EMT-associated genes in MCF10A cells, confined (for 6 h and 24 h) versus unconfined, measured by RT-qPCR (mean ± SD; n = 3 independent experiments). **(e)** Bright field images showing directed migration of MCF10A cells confined for 48 h with glass LifterslipsTM manufactured with 50 µm-thick spacers. This experiment demonstrates that the coverslip pressure was not responsible for outwards migration. NC: unconfined; +C: confined. Scale bar, 500 µm.

**Supplementary Figure 2**


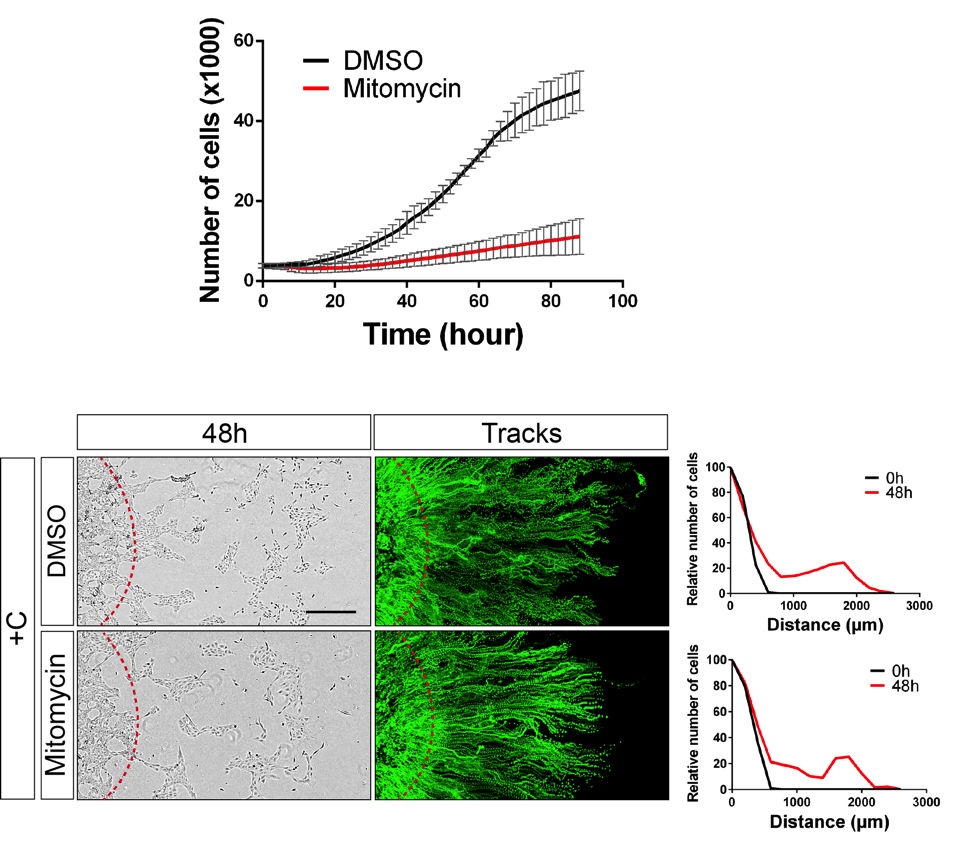


**a**

**b**

**O_2_-directed cell migration does not depend on cell proliferation**

**(a)** Proliferation curves of the H2B-GFP labelled MCF10A cells treated with the inhibitor of cell proliferation mitomycin or vehicle only (DMSO) (mean number of cells/image ± SD; n = 3 independent experiments). This experiment was performed with the Incucyte imaging system. **(b)** Tracking and redistribution of H2B-GFP-expressing MCF10A at 48 h post-confinement following treatment with mitomycin or vehicle only. In both experiments, cells pre-treated for 3 h with mitomycin (3 µM) or vehicle were washed with mitomycin-free medium before confinement was applied. +C: confined. Scale bar, 500 µm.

**Supplementary Figure 3**

| **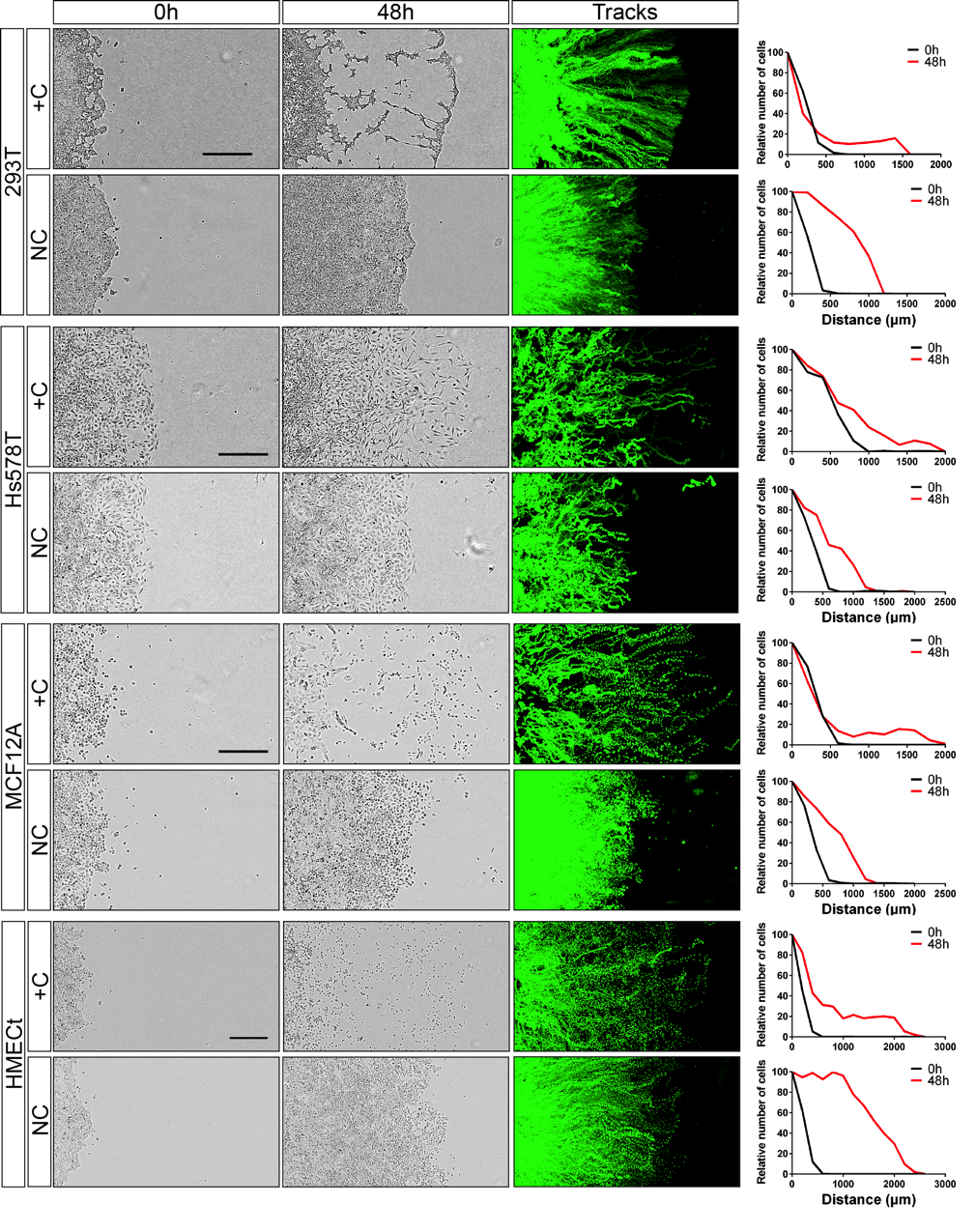** |
| --- |

**Aerotaxis is shared by various epithelial cell lines**. Directed migration and redistribution of different epithelial cell lines under confinement or unconfined including 293T, Hs578T, MCF12A, and HMECt (also see Supplementary Movies 4-7). NC: unconfined; +C: confined. Scale bar, 500 µm.

**Supplementary Figure 4**

| **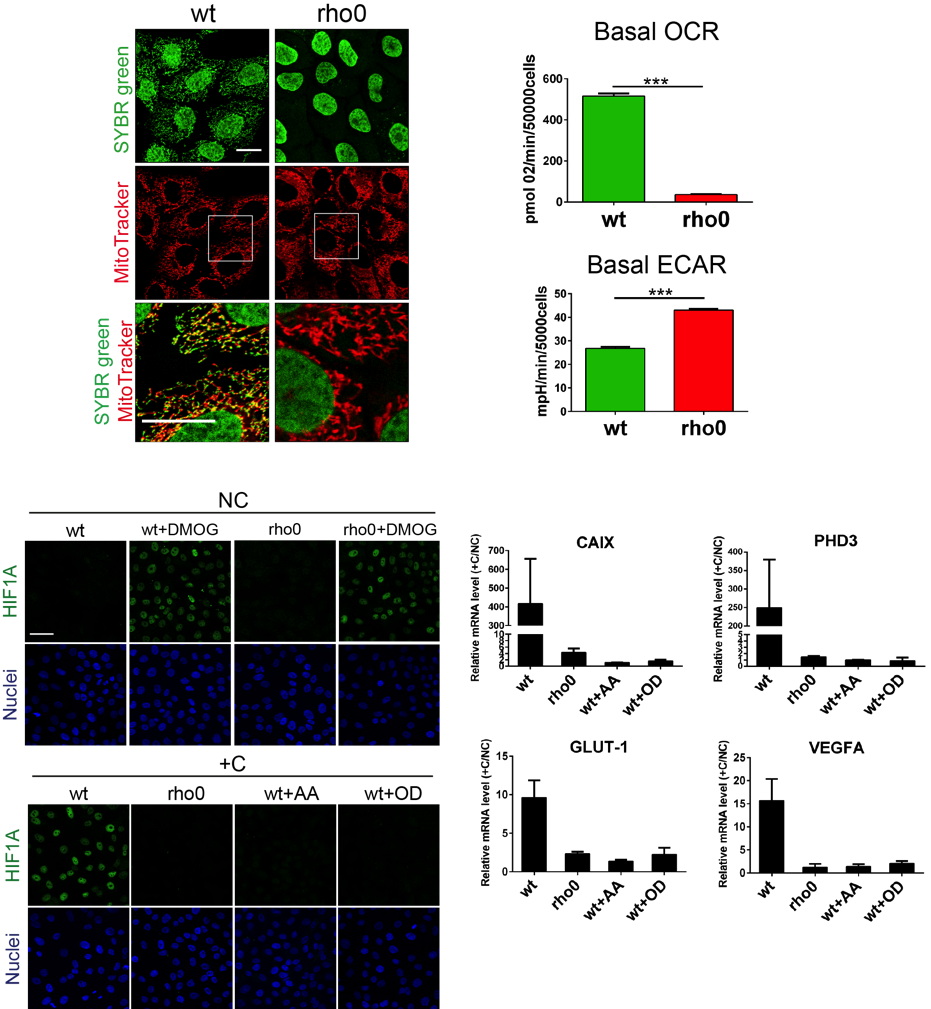**  **c**  **a**  **b**  **d** |
| --- |

**Self-generation of hypoxia under confinement relies on mitochondrial O_2_ consumption**

**(a)** Depletion of mitochondrial DNA in rho0 cells visualised by SYBR green labelling (green). Mitochondria were further stained with Mito-tracker red. Lower panels represent merged and enlarged views of the framed regions. This experiment demonstrates that mitochondria of rho0 cells are devoid of mtDNA. Scale bar, 20 µm **(b)** Computational analysis of the data obtained in Fig. 2b. Basal OCR (oxygen consumption rate) and basal ECAR (extracellular acidification rate) are given as the mean ± SD of five different wells at three different times. Rho0 cells were unable to respire and compared to wt cells, the acidification rate of their medium was higher due to increased glycolysis. **(c)** Immunofluorescence staining of HIF1A showing that cells with an altered electron transport chain can not generate hypoxia under confinement, even though they may still accumulate HIF1A after inhibition of PHDs. Upper panels: HIF1A stabilization in MCF10A and rho0 cells treated with the PHD inhibitor DMOG. Lower panels: confined rho0 or wt-MCF10A cells treated with antimycin A (AA 0.5 µM) and oligomycin D (OD 0.25 µM) are no longer able to create hypoxia and to stabilize HIF1A. Scale bar, 40 µm. **(d)** Relative expression of HIF1A target genes measured by RT-qPCR, in wt, rho0 or wt MCF10A cells treated with AA (0.5 µM) or OD (0.25 µM) and confined for 24 hours vs unconfined ) (mean ± SD; n = 3 independent experiments). NC: unconfined, +C: confined. *** P < 0.001 by two-tailed Student’s t-test.

**Supplementary Figure 5**

| **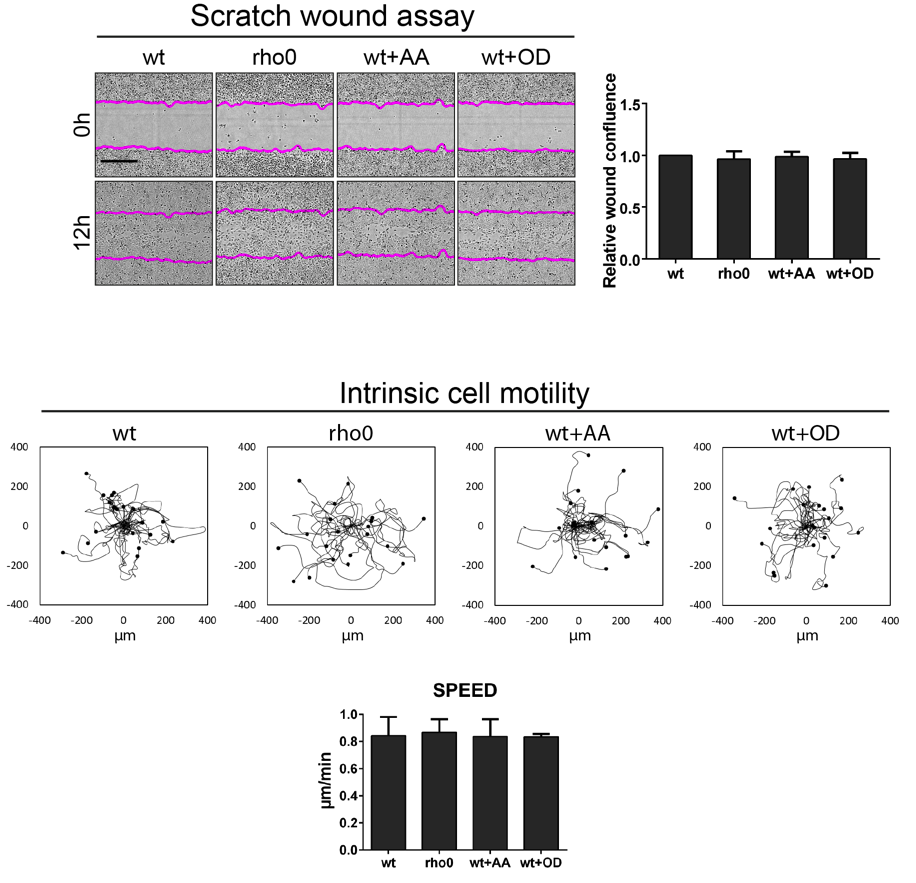**  **b**  **a** |
| --- |

**Functional mitochondria are not required for intrinsic cell motility**

**(a)** Cell migration analysis and quantification with the IncuCyte™ scratch wound migration assay of MCF10A, rho0 and MCF10A cells treated with AA (0.5 µM) or OD (0.25 µM). Scale bar, 500 µm. Relative wound confluence: mean ± SD; n = 3 experiments. **(b)** Intrinsic motility analysis of isolated cells seeded at 5% density. Individual cell tracking was performed for wt , rho0 and wt MCF10A cells treated with AA (0.5 µM) or OD (0.25 µM) for 12 h (20 cells per experiment; n = 3). Speed values were calculated from individual cell tracks and represented as bar charts (mean ± SD; n = 3 experiments).

**Supplementary Figure 6**

**b**


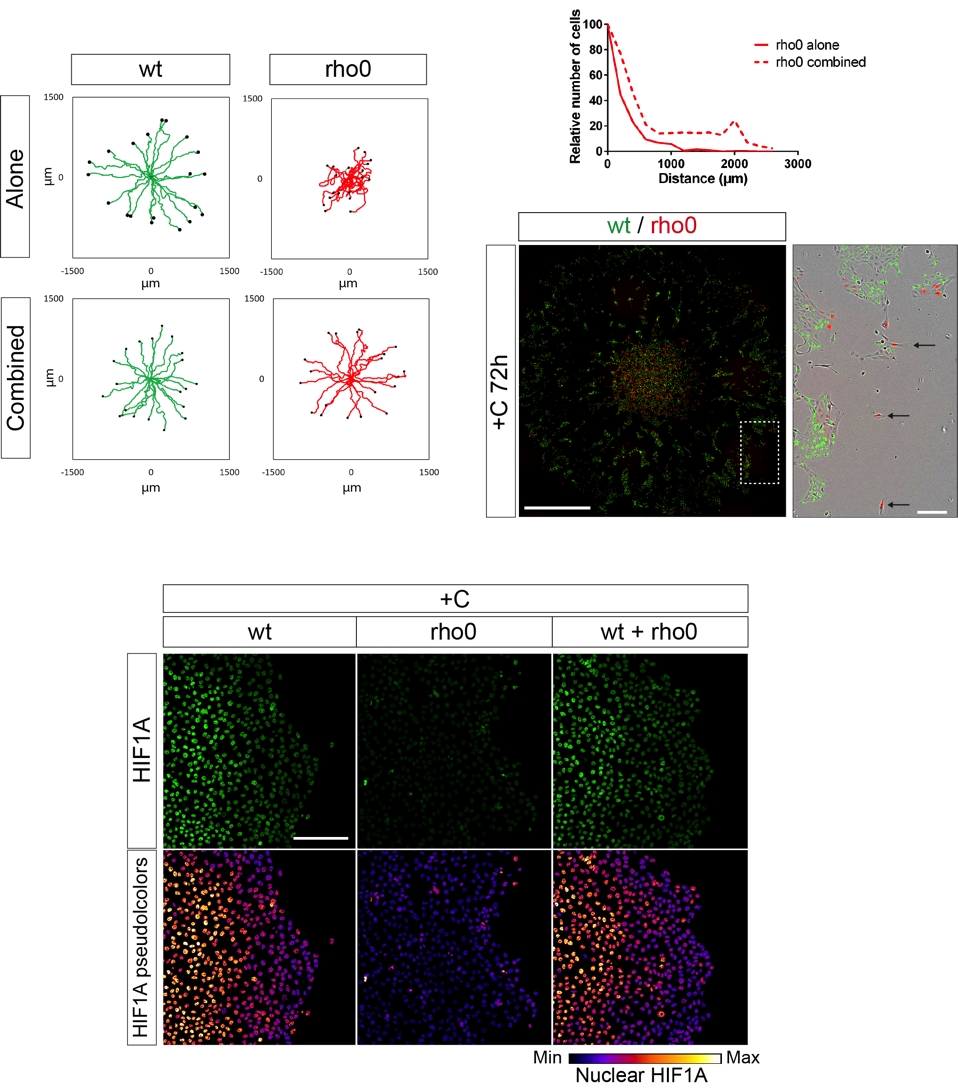


**d**

**c**

**a**

**Rho0 cells demonstrate aerotactic skills, provided that an oxygen gradient is created by wt cells**

**(a)** Individual tracking of 20 wt and rho0 cells seeded alone or combined (from experiments depicted in Fig. 2d). **(b)** Redistribution at 48 h post-confinement of rho0 cells alone or combined with wt cells, showing increased directional migration of rho0 cells in the combined condition. **(c)** Fluorescence imaging of cell dispersion of combined wt (green) and rho0 (red) cells 72 h post-confinement demonstrating aerotactic capacities of rho0 cells when combined with wt cells (also see Supplementary Movie 8). Isolated rho0 cells can be found at the migration front (black arrows on the high magnification panel) suggesting that they have not migrated “piggy backed” with wt cells. Scale bars, 2 mm (left panel), 200 µm (right panel) **(d)** A gradient of HIF1A stabilization is observed 3 h after confinement with wt or wt+rho0 cells (ratio 1:1) but not with rho0 cells only. HIF1A protein level detected by immunofluorescence displayed in green (upper panels) or in pseudocolors (lower panels) revealed the hypoxic gradient located at the edge of the cell cluster. Scale bar, 200 µm.

**Supplementary Figure 7**


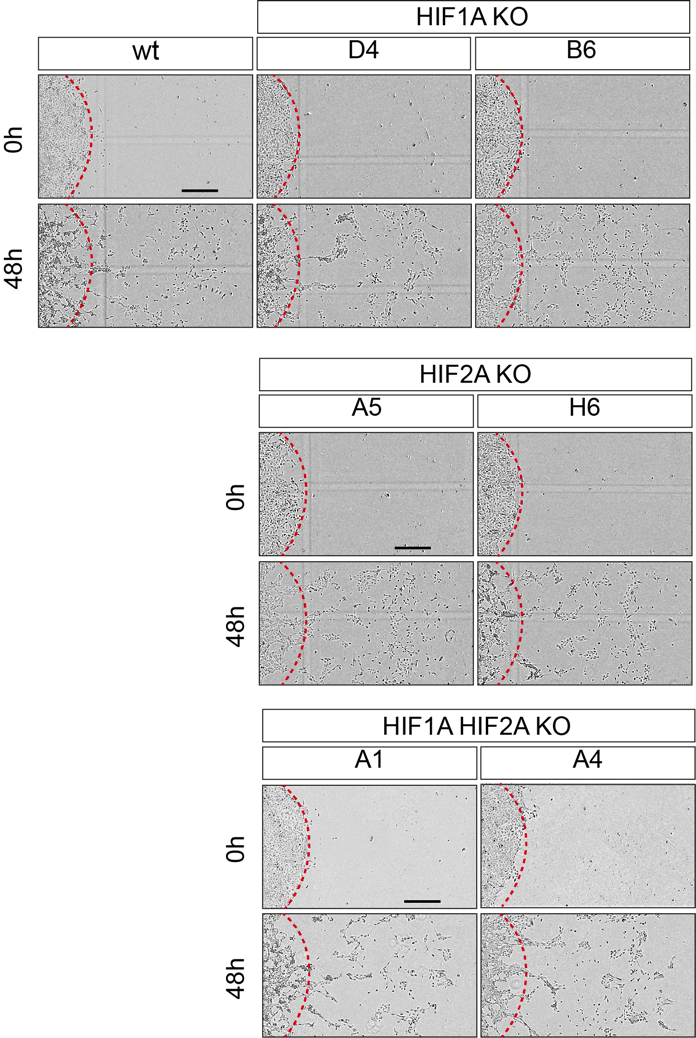


**HIFs Factors are dispensable for aerotaxis**

Bright field images showing directed migration of the *HIF1A, HIF2A* or *HIF1A+HIF2A* knock-out MCF10A clones presented in Fig. 3a, 3b and 3c. Scale bar, 500 µm.

**Supplementary Figure 8**

**b**

**a**

| 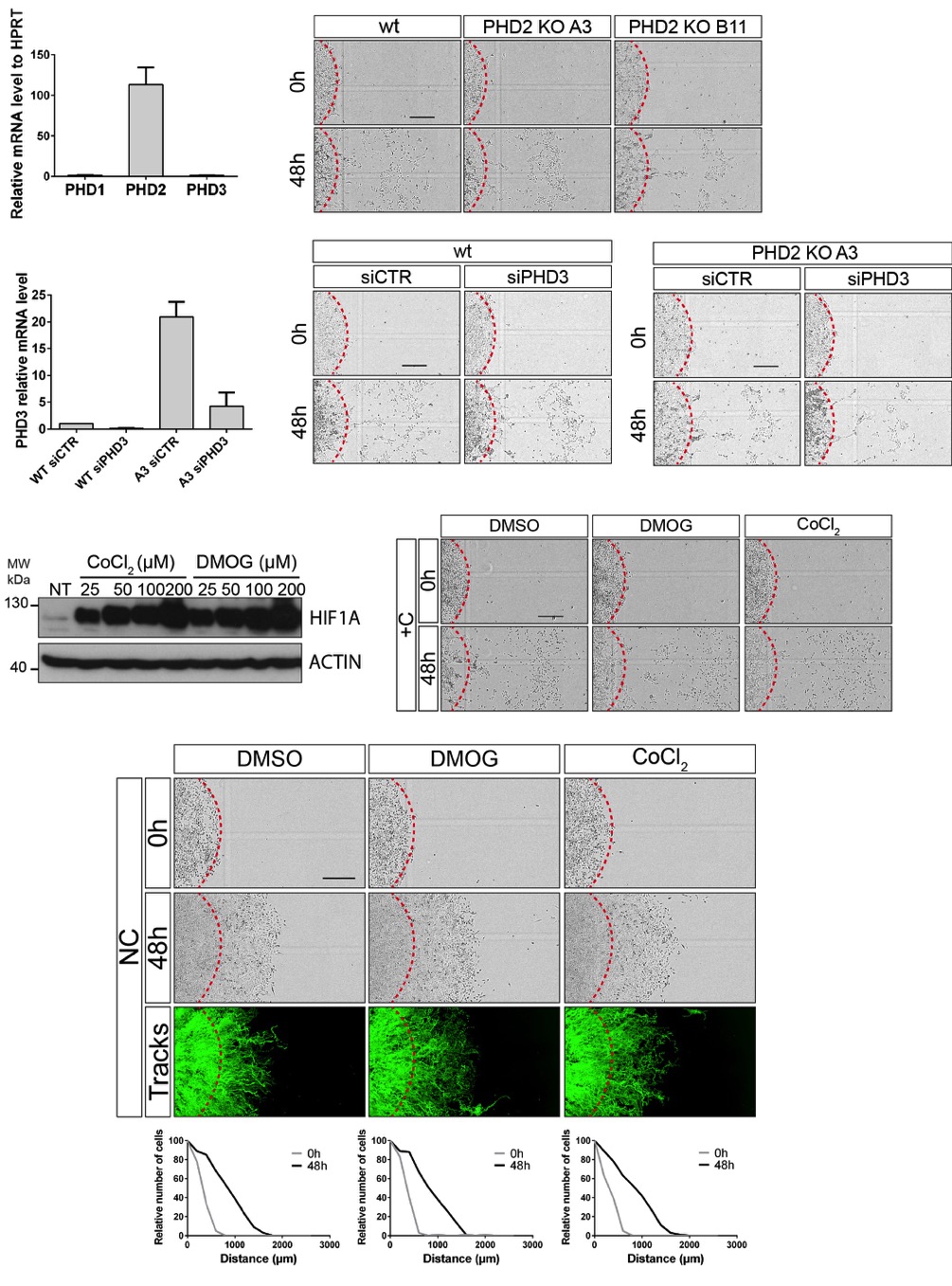  **h**  **c**  **e**  **d**  **f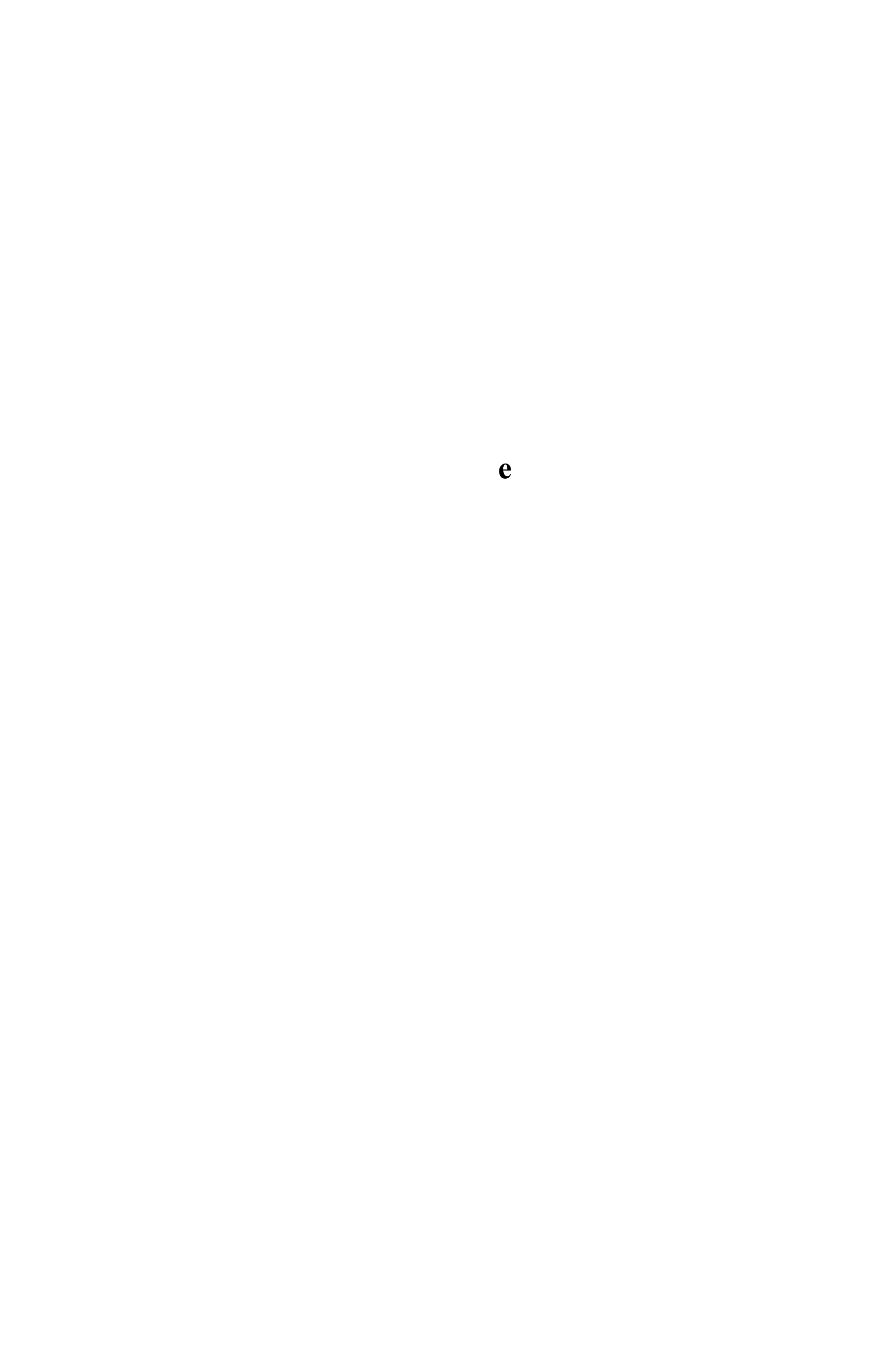**  **g**   \|  \| \| --- \| |
| --- | --- |

**PHDs are not required for aerotaxis**

**(a)** *PHDs* mRNA expression in MCF10A cells analysed by RT-qPCR (mean ± SD; n= 3 independent experiments). **(b, d, e)** Bright field images showing the directed migration under confinement of the indicated MCF10A cells, *i.e.* *PDH3* KO clones, and either wt cells or a *PDH3* KO clone silenced for *PHD2*, corresponding respectively to the experiments depicted in Fig. 3d, 3e and 3f. Scale bar, 500 µm. **(c)** Validation of *PHD3* silencing in wt cells and the *PHD2* KO clone A3 by RT-qPCR (mean ± SD; n= 3 independent experiments). **(f)** Immunoblot showing HIF1A protein accumulation in MCF10A cells on DMOG and CoCl_2_ treatment for 4 h in normoxia. **(g)** Bright field images showing directed migration under confinement of MCF10A cells treated with DMOG (50 µM) and CoCl_2_ (50 µM) corresponding to the experiments depicted in Fig. 3g. Scale bar, 500 µm. **(h)** Tracking and redistribution of unconfined MCF10A expressing H2B-GFP treated with DMOG (50 µM) or CoCl_2_ (50 µM) demonstrating that PHDs inhibition, and the resulting HIFA stabilisation, does not induce cell migration in normoxic condition. NC: unconfined; +C: confined. Scale bar, 500 µm.

**Supplementary Figure 9**

**a**

| **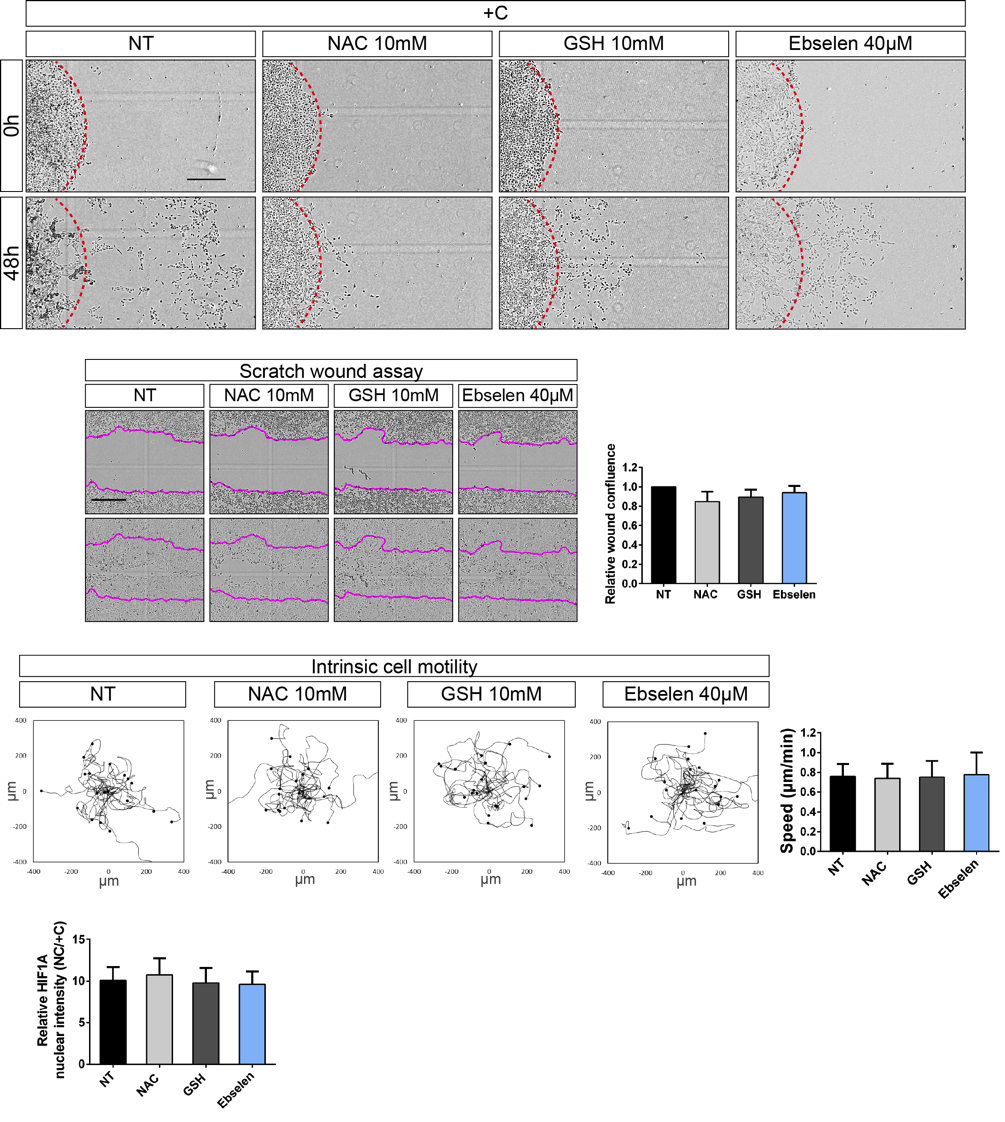**  **d**  **b**  **c** |
| --- |

**Antioxidants inhibit aerotaxis but neither intrinsic cell motility, nor hypoxia generation**

**(a)** Bright field images showing impaired directed migration of MCF10A cells under confinement (+C) by antioxidants NAC (10 mM), GSH (10 mM) or Ebselen (40 µM) corresponding to the experiments depicted in Fig. 4a. Scale bar, 500 µm **(b)** Scratch wound assay of MCF10A cells not treated (NT) or treated with NAC (10 mM), GSH (10 mM) or Ebselen (40 µM), showing no effect of these antioxidants on cell motility. Experiments were recorded and analysed with the IncuCyte™ scratch wound assay. Quantification of the relative wound confluence is given (mean ± SD; n = 3 independent experiments). Scale bar, 500 µm. **(c)** Intrinsic cell motility of isolated MCF10A cells seeded at 5% confluence is not affected by the antioxidants NAC (10 mM), GSH (10 mM) or Ebselen (40 µM) (mean ± SD; n = 3 experiments). **(d)** NAC (10 mM), GSH (10 mM) or Ebselen (40 µM) do not compromise the ability of cells to generate hypoxia as demonstrated by a ten-fold induction of the HIF1A immunofluorescence signal intensity after 6 h of confinement (mean ± SD; n=3 independent experiments). +C: confined; NT: Not treated.

**Supplementary Figure 10**

**a**

| 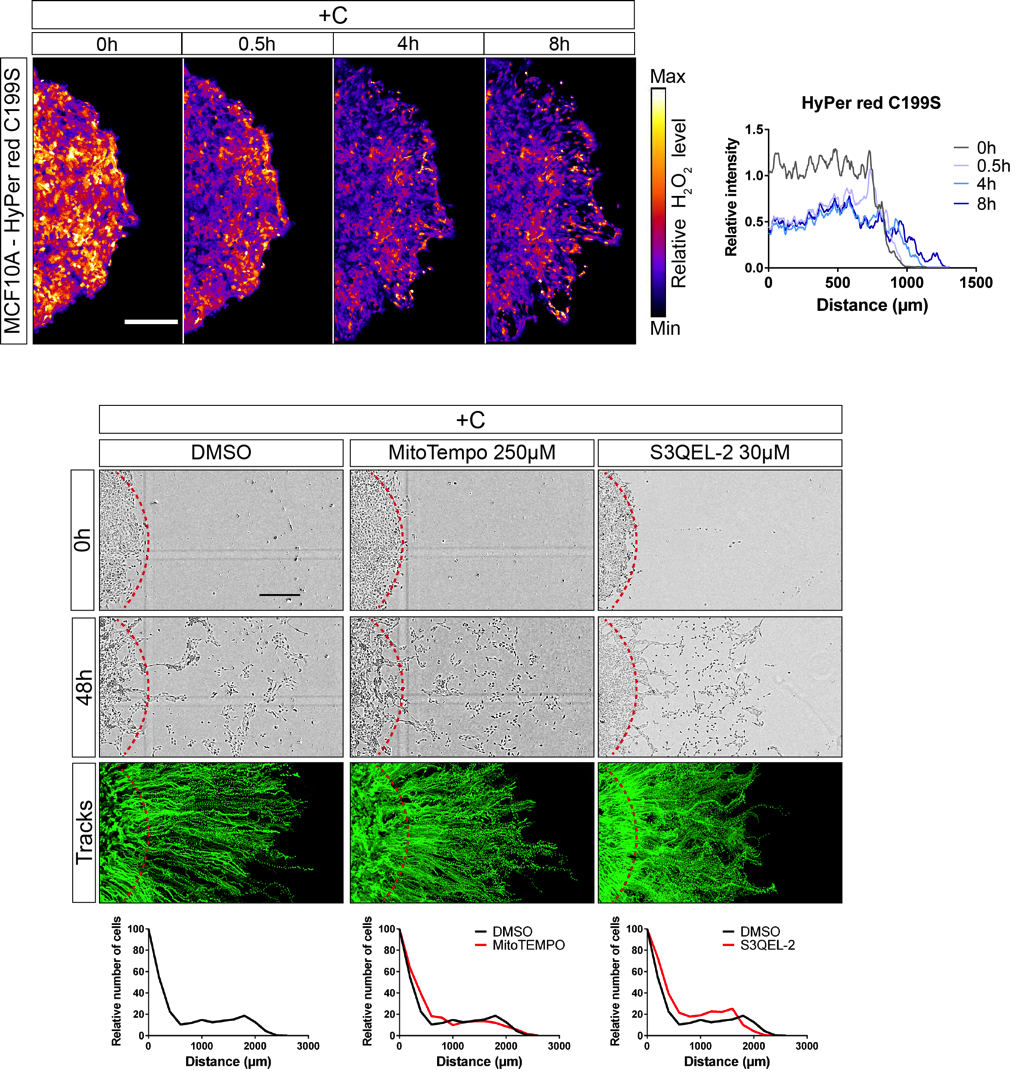  **b** |
| --- |

**ROS generation and aerotaxis**

**(a)** A homogeneous drop of pH, represented in pseudocolors, consecutive to cell adaptation to hypoxia, was detected with the pH sensitive but ROS insensitive probe HyPer red (C199S) following confinement for 0 h, 0.5 h, 4 h and 8 h. Experiment was performed with cells co-expressing the fluorescent probe HyPer red (C199S) and the ROS sensitive probe HyPer-3, the signal of the latter being displayed in Fig. 4e. Scale bar, 500 µm. A graph representing the HyPer red (C199S) fluorescence intensity at 540 nm across the radius of the cell cluster is also provided (right panel). **(b)** Tracking and redistribution of H2B-GFP-expressing MCF10A cells at 48 h post-confinement following treatment with the mitochondria-targeted ROS scavengers MitoTEMPO (250 µM) and S3QEL-2 (30 µM), showing that mitochondrial ROS are not involved in aerotaxis. Scale bar, 500 µm.

**Supplementary Figure 11**

| 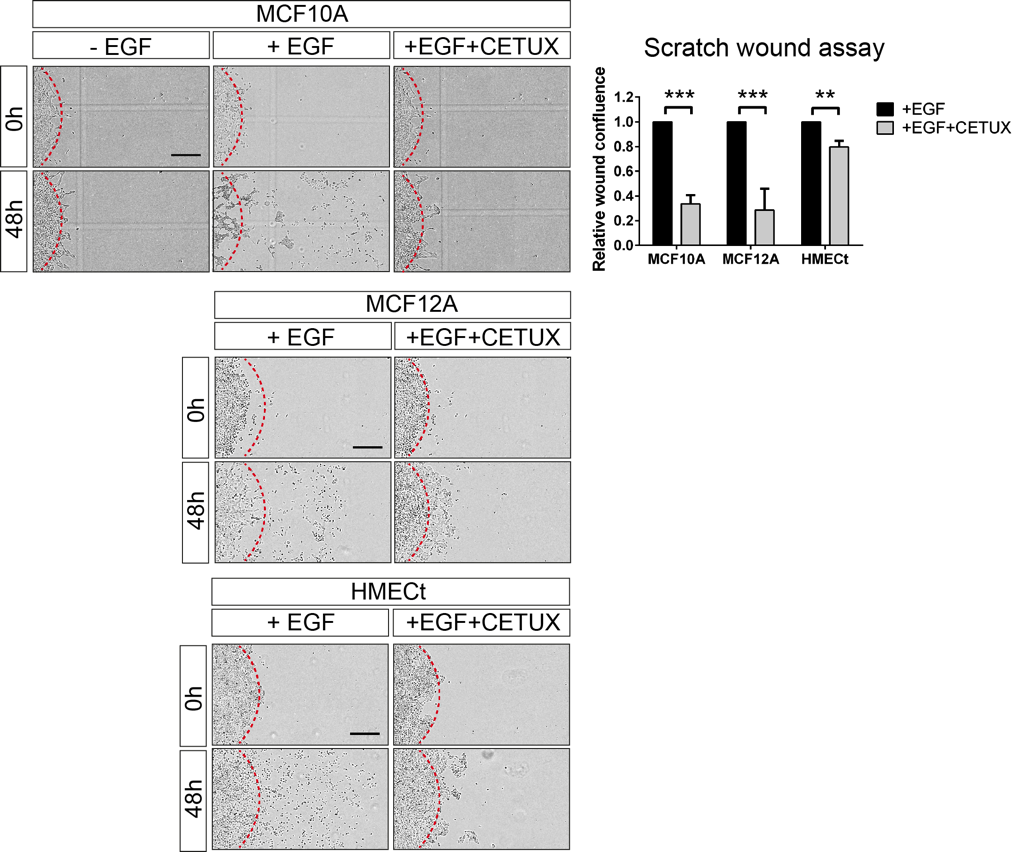  **b**  **a** |
| --- |

**Inhibition of EGFR activity by Cetuximab impairs O_2_-directed migration**

**(a)** Bright field images corresponding to the experiments presented in Fig. 5a show that inhibition of EGFR signalling in MCF10A, MCF12A and HMECt cells abolishes aerotaxis. Scale bar, 500 µm. **(b)** The motility of MCF10A, MCF12A and HMECt cells treated or not with Cetuximab (25 µg.mL^-1^) was analysed with the IncuCyte™ scratch wound assay (mean ± SD; n = 3 independent experiments). This experiment demonstrates that inhibition of EGFR signalling by Cetuximab in HMECt cells does not prevent their motility. *** P < 0.001, ** P < 0.01 by two-tailed Student’s t-test.

**Supplementary Figure 12**

| 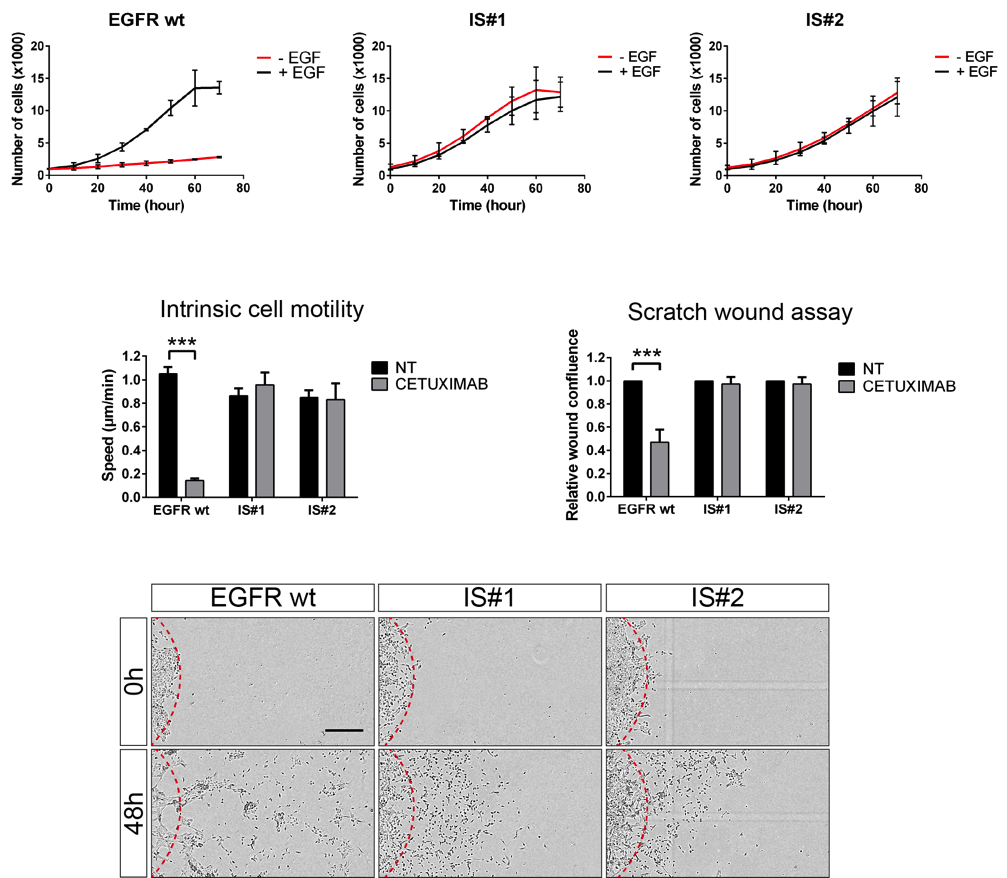  **d**  **c**  **b**  **a** |
| --- |

**Constitutive and EGF-independent activation of EGFR impairs aerotaxis but not cell motility**

*IS#1* and *IS#2* clones are H2B-GFP labelled MCF10A cells expressing a constitutively activated EGFR mutant and are invalidated for the endogenous *EGFR* gene (see Fig.5d). **(a)** Growth curves demonstrate that *IS* clones proliferate independently of EGF (mean number of cells/image ± SD). **(b)** Intrinsic cell motility of isolated MCF10A cells expressing *wt EGFR* or , *IS#1* and *IS#2* clones seeded at 5% confluence (mean ± SD; n = 3 experiments). **(c)** Cell migration analysis of IS clones versus MCF10A cells expressing *wt EGFR*, performed with the IncuCyte™ scratch wound assay (mean ± SD; n = 3 independent experiments). These experiments show that intrinsic cell motility of *IS#1* and *IS#2* clones is EGF-independent. **(d)** Bright field images corresponding to the experiment presented in Fig. 5e, with *IS#1* and *IS#2* clones showing the absence of the characteristic migration front observed with wt cells. Scale bar, 500 µm. *** P < 0.001 by two-tailed Student’s t-test. NT: Not treated.

**Supplementary Figure 13**


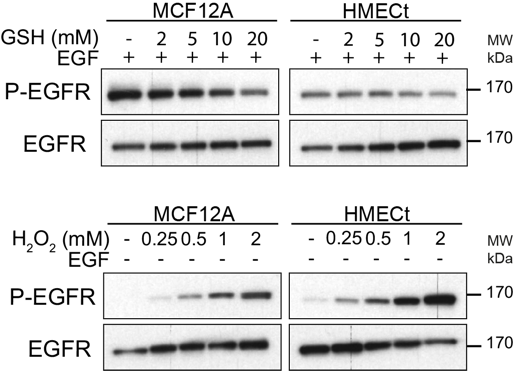


**Redox regulation of EGFR activity in epithelial cell lines**

Immunoblots showing respectively inhibition and activation of EGFR phosphorylation (Y1173) by GSH and H_2_O_2_ in MCF12A and HMECt epithelial cell lines (EGF 5 ng.mL^-1^).

**Supplementary Figure 14**

| 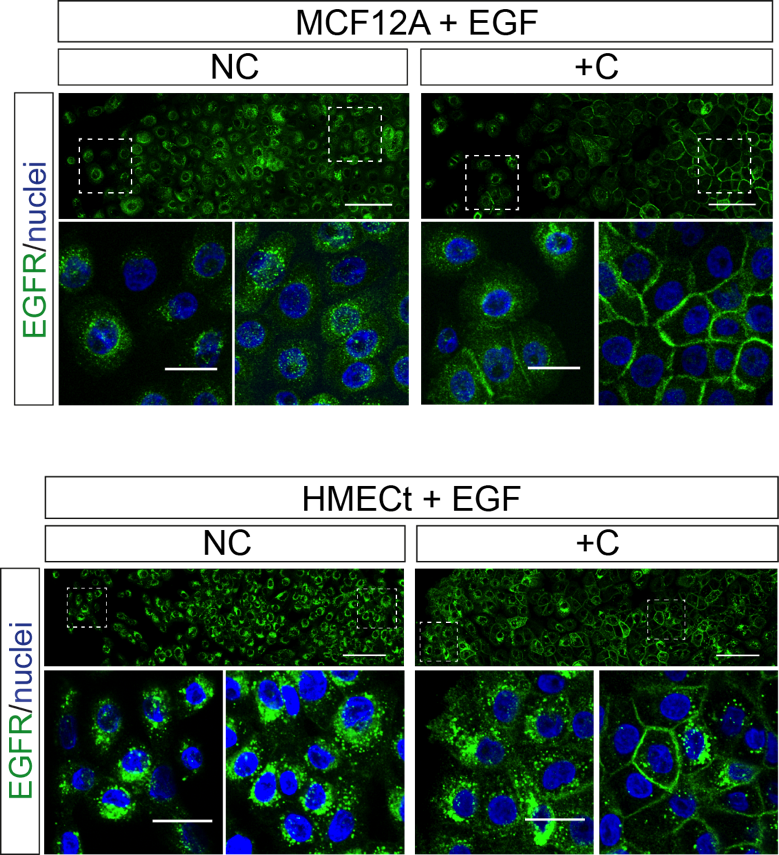 |
| --- |

**A gradient of EGFR activation is observed under confinement**

Immunofluorescence images of MCF12A or HMECt cell clusters. EGFR activation is visualised by cytoplasmic green dots corresponding to the internalisation of the phosphorylated receptor. Non-activated EGFR is localised at the plasma membrane. The lower panels display enlarged views of the framed regions at the edge and centre of the clusters. IF was performed with total EGFR antibody (green). Nuclei are stained with DAPI (blue). Images are representative of 3 experiments. These results show that under confinement, EGFR is only activated in the cells at the edge of the cluster. NC: unconfined; +C: confined for 6 h. Scale bars, 100 µm (top), 30 µm (bottom).

**Supplementary Figure 15**

| 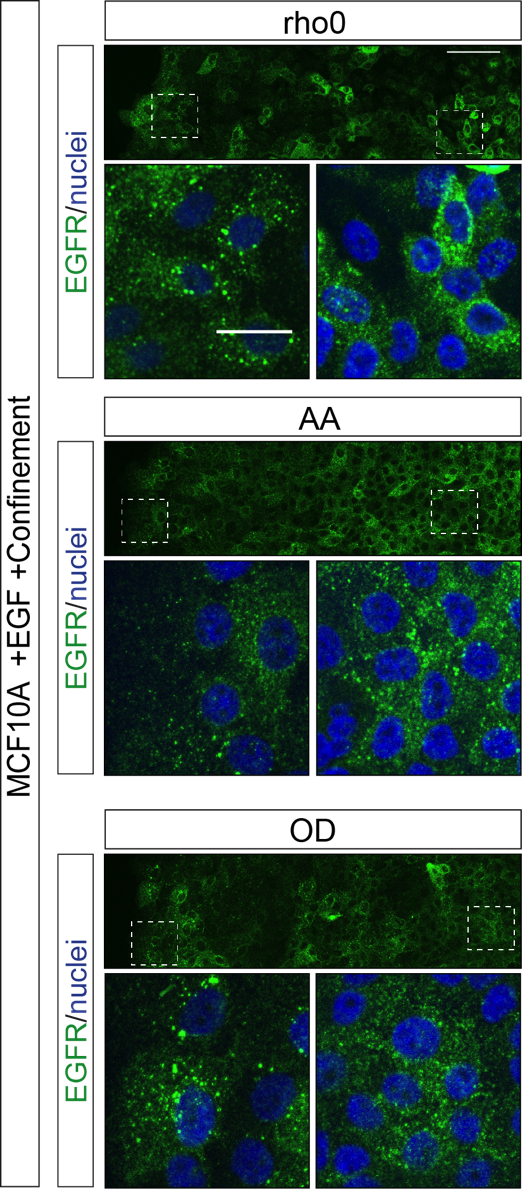 |
| --- |

**The gradient of EGFR activity observed under confinement depends on the capacity of cells to generate hypoxia**

Immunofluorescence images showing EGFR activation (visualised by cytoplasmic green dots) along the radius of the cell clusters (rho0 or MCF10A treated with AA (0.5 µM) or OD (0.25 µM)) (to be compared with experiments shown in Fig. 6d). The lower panels for each cell line display enlarged views of the framed regions at the border or centre of the clusters. IF was performed with total EGFR antibody (green). Nuclei are stained with DAPI (blue). Images are representative of 3 experiments. These results demonstrate that in the absence of hypoxia, EGFR is activated all along the radius of the cell clusters. Cells were confined for 6 h. Scale bar, 100 µm (top), 30 µm (bottom).

**Supplementary Figure 16**

**Uncropped WB**

**
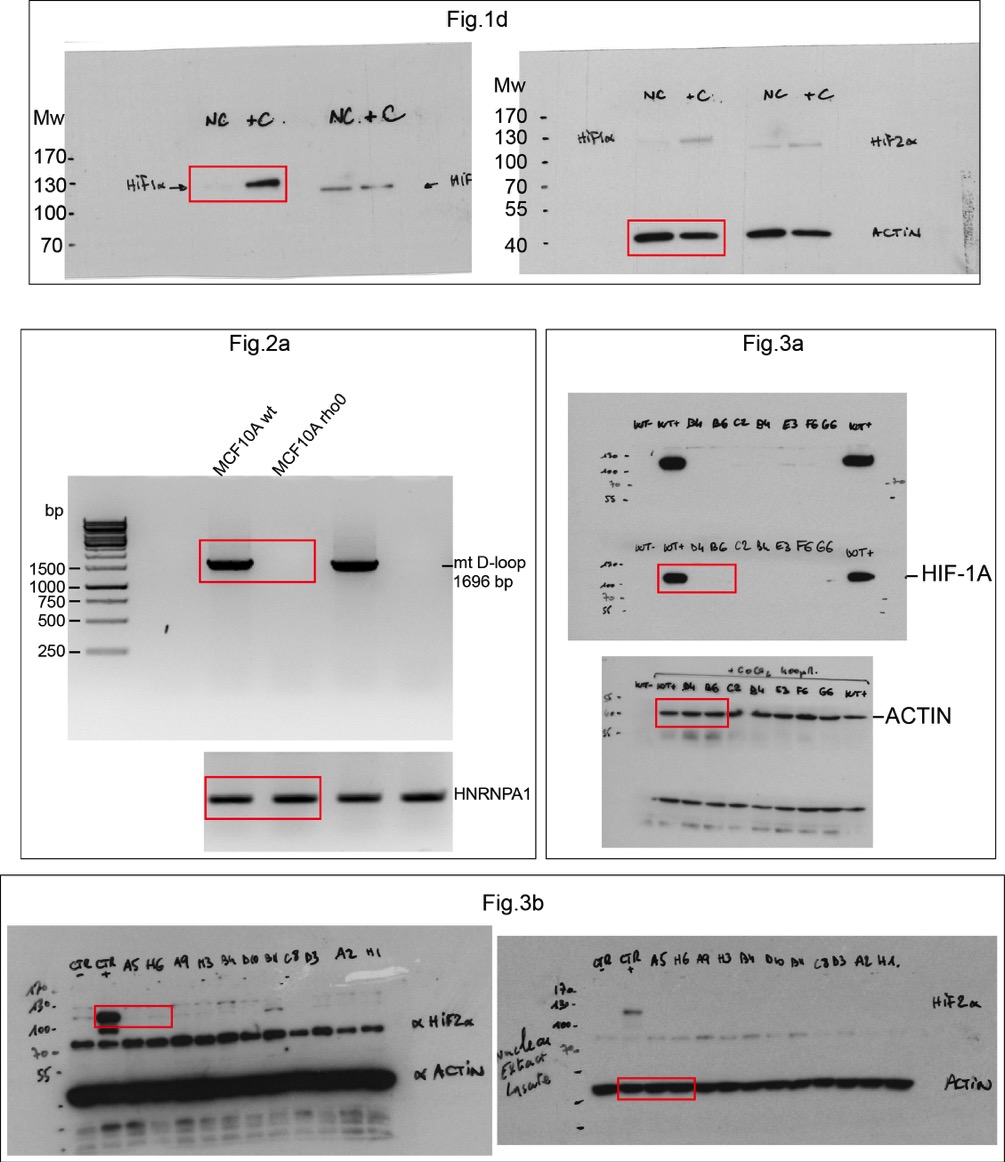
**


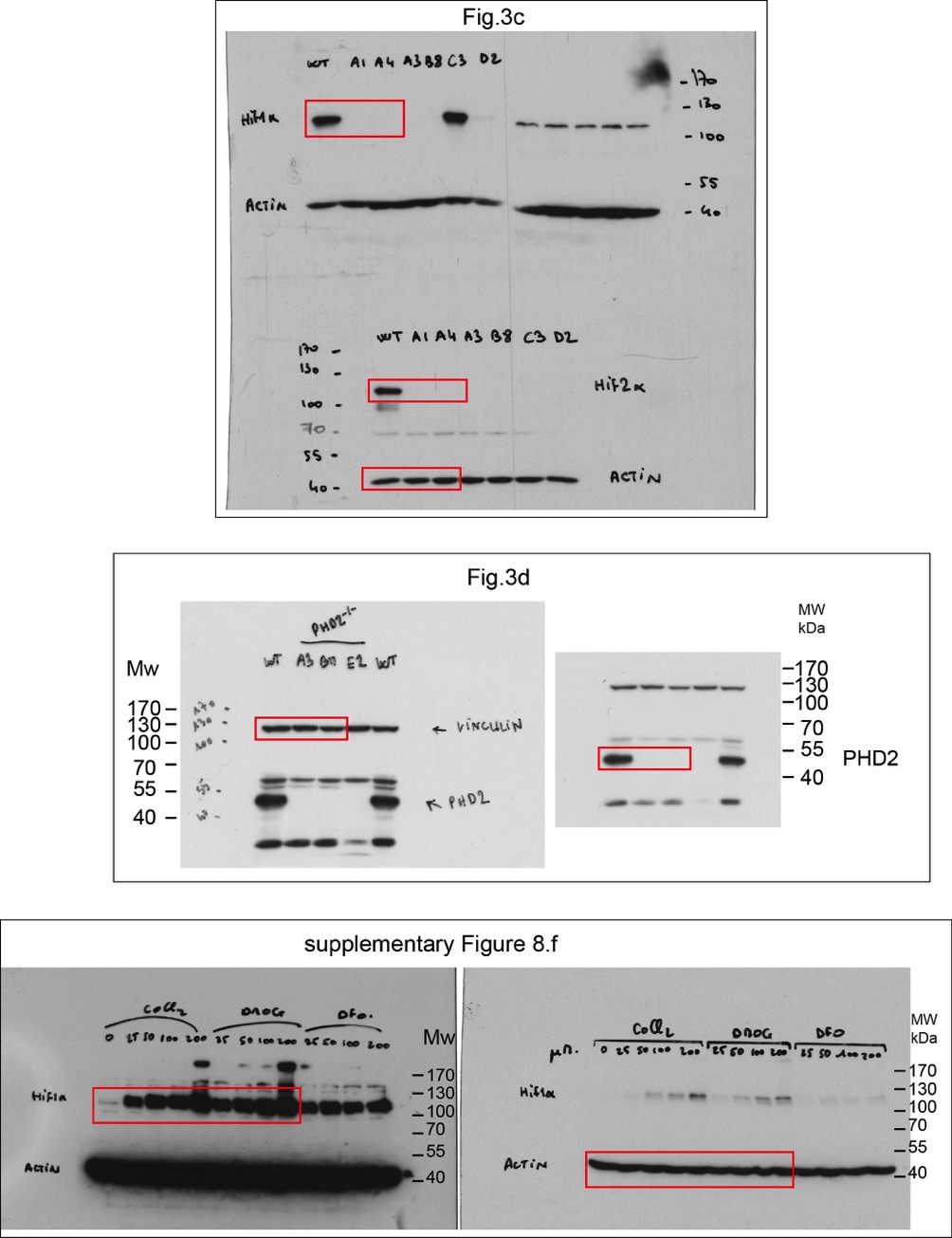


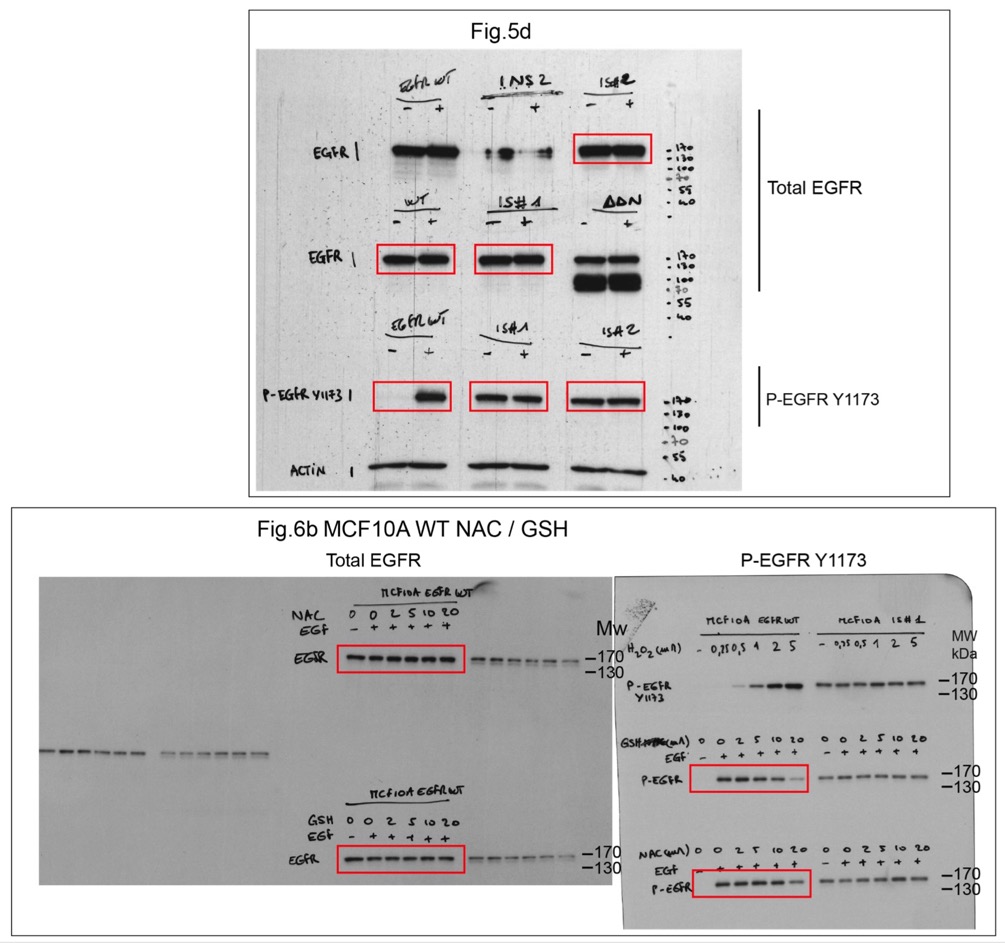


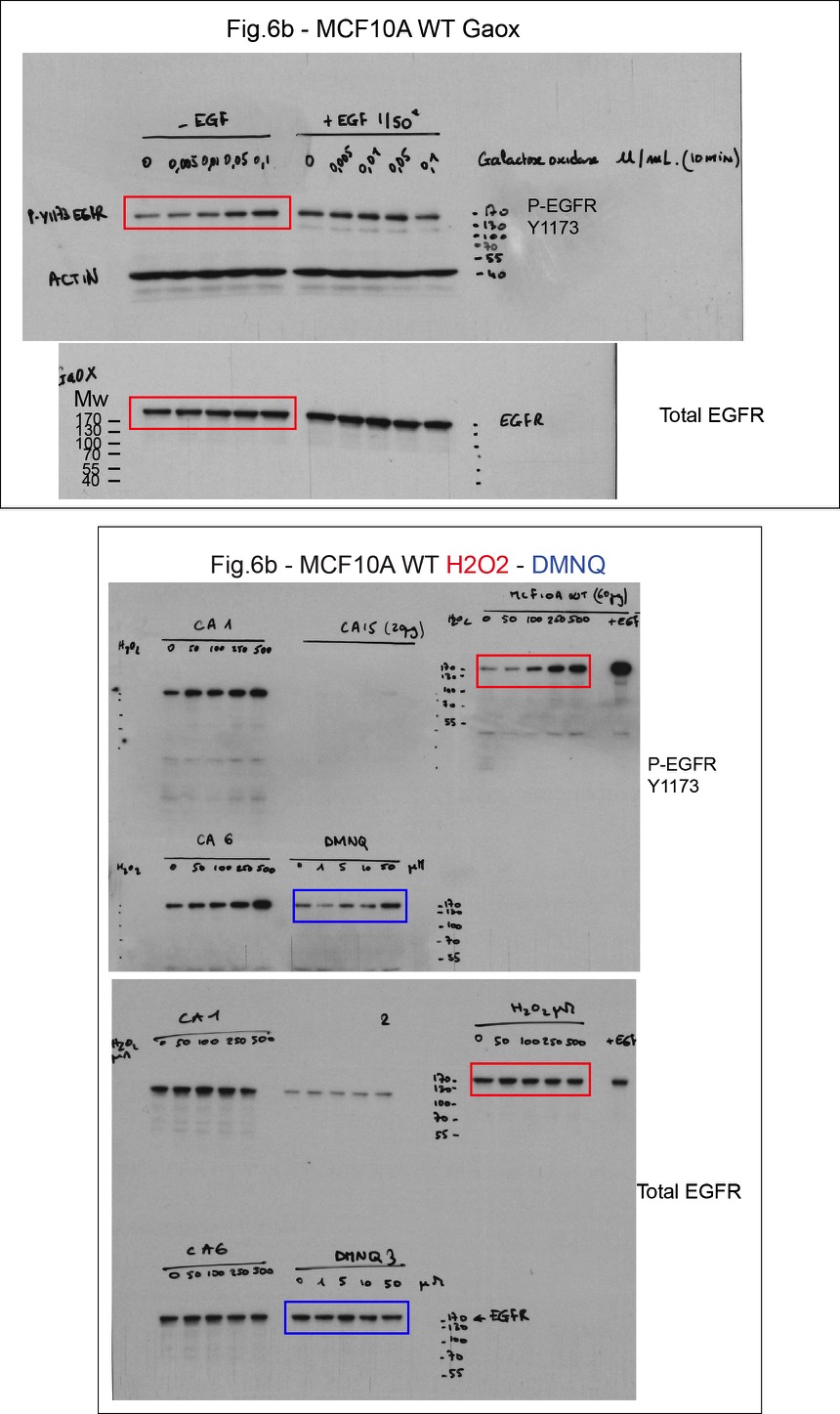


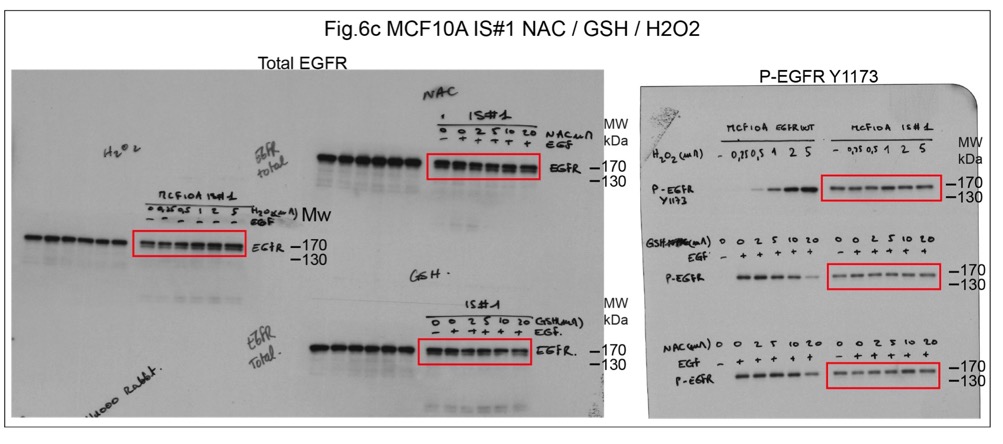


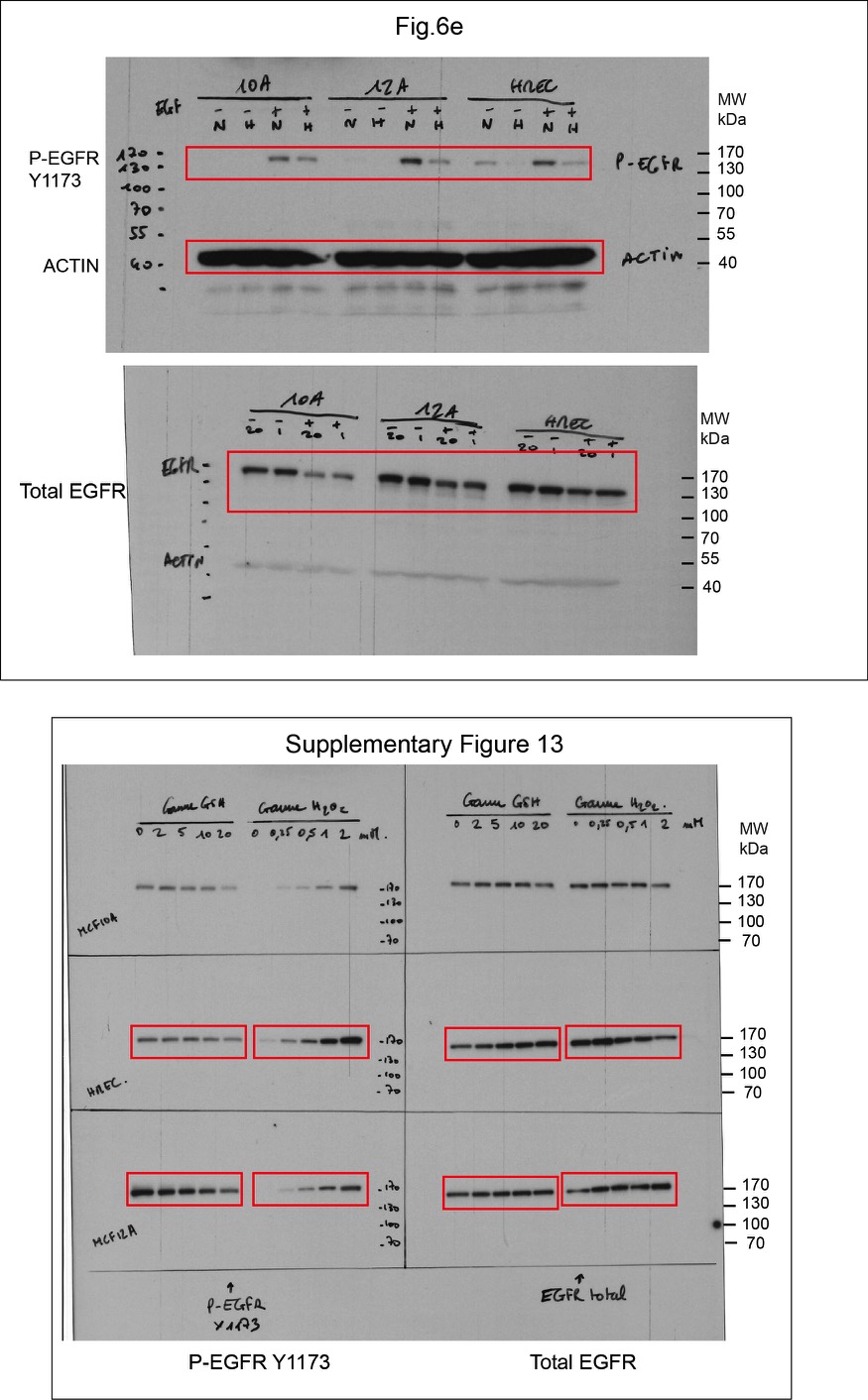

Supplement: Supplementary file 1 — Supplementary Information [file 41467_2018_6988_MOESM1_ESM.docx]
