# Supplementary material for: Redox regulation of EGFR steers migration of hypoxic mammary cells towards oxygen
Source: Nat Commun. 2018 Oct 31;9:4545. doi: 10.1038/s41467-018-06988-3 (PMC6208388; doi:10.1038/s41467-018-06988-3)
Supplement: Supplementary file 2 — Description of Additional Supplementary Files [file 41467_2018_6988_MOESM2_ESM.pdf]

Legends of the Supplementary Movies are as follow :

- **Supplementary Movie 1**: Time-lapse video (1 h) representing hypoxia generation following MCF10A cell confinement using a porphyrin-based oxygen measurement system (Visisens) (related to Fig. 1c).
- **Supplementary Movie 2**: Combined time-lapse video representing a whole cluster of H2B-GFP MCF10A confined (+C) or not (NC) for 48 h (related to Fig. 1f).
- **Supplementary Movie 3**: Combined time-lapse videos representing directional migration of MCF10A cells under confinement (+C) or not (NC) for 48 h. Scale bar 500 µm (related to Fig. 1h). Note that only the cells at the front migrate directionally whereas those located behind the front wave are moving randomly.
- **Supplementary Movie 4**: Combined time-lapse videos (48 h) representing directional migration of 293T, MCF12A, Hs578T, and HMECt cells under confinement demonstrating that aerotaxis is not restricted to MCF10A cells (related to Supplementary Fig. 3). Scale bar 500 µm.
- **Supplementary Movie 5**: Combined time-lapse videos representing directional migration under confinement of wt cells (green) alone (left panel), non-directional motility of rho0 cells (red) alone (middle panel), and directional migration of rho0 cells when combined with wt cells (right panel)(related to Fig. 2d). Rho0 cells undergo aerotaxis when placed in a hypoxia gradient generated by wt cells, although they do not use oxygen for their energy metabolism.
- **Supplementary Movie 6**: Time-lapse video depicting generation after confinement of a H_2O_2 gradient at the cell cluster periphery detected by the fluorescent probe HyPer-3 (related to Fig. 4e). A ROS burst concomitant to the creation of the oxygen gradient can be observed at the edge of the cell cluster (see Supplementary Movie 1 for comparison).
- **Supplementary Movie 7**: Combined time-lapse videos (48h) representing random migration of MCF10A *IS#1* cells under confinement compared to wt cells (related to Fig. 5e). Scale bar 500 µm.
- **Supplementary Movie 8**: Combined time-lapse videos representing the sustained formation of lamellipodia (imaged by Lifeact-mCherry expression) at the leading edge of MCF10A cells migrating under confinement (+C) and their random generation for unconfined cells (NC) (related to Fig. 7).
